# Circadian Regulation of Glucose Metabolism: Implications for Pathogenesis and Chronotherapy of Type 2 Diabetes

**DOI:** 10.3390/diabetology7070122

**Published:** 2026-06-26

**Authors:** Michael Oraebosi, Connor Baucom, Ruifeng Cao

**Affiliations:** 1 Department of Neuroscience and Cell Biology, Robert Wood Johnson Medical School, Rutgers University, Piscataway, NJ 08854-0009, USA;; 2 Department of Neurology, Robert Wood Johnson Medical School, Rutgers University, Piscataway NJ, 08854-0009, USA

**Keywords:** circadian rhythm, diabetes, glucose metabolism, glycemic control

## Abstract

The global prevalence of type 2 diabetes continues to rise at an alarming pace, challenging existing strategies for disease prevention and management. Beyond traditional risk factors, increasing evidence indicates that glucose metabolism is temporally regulated by the body’s 24 h biological clock and oscillates based on the time of day. Disturbances of the circadian clock function are linked to impairments in glucose homeostasis and increased risk of obesity and diabetes. This review explores the intricate relationship between the circadian system and glucose homeostatic control. We begin with an introduction to the hierarchical organization of the circadian system. Next, we examine the role of the circadian clock in regulating organs and tissues that are involved in glucose metabolism, i.e., the pancreas, skeletal muscles, the liver and adipose tissue. We next review evidence that supports the involvement of circadian disturbances in the pathogenesis of diabetes. Finally, we discuss chronotherapy and its potential application in clinical intervention of diabetes. As type 2 diabetes becomes increasingly common worldwide, understanding how the body’s internal clock shapes this disease may open new and powerful opportunities for its prevention and treatment.

## 1. Introduction

Diabetes mellitus is a disorder of carbohydrate, fat, and protein metabolism and is characterized by persistent hyperglycemia. It is broadly classified into type 1 diabetes, type 2 diabetes, gestational diabetes, and other specific forms such as monogenic and secondary diabetes. Type 1 diabetes results from autoimmune destruction of pancreatic β-cells, leading to absolute insulin deficiency, whereas type 2 diabetes is characterized by insulin resistance combined with progressive β-cell dysfunction. Gestational diabetes occurs during pregnancy and is associated with both short- and long-term metabolic consequences for the mother and offspring. Clinically, patients are diagnosed with diabetes mellitus by fasting blood glucose (FBG) of ≥7.0 mmol/L (126 mg/dL); random blood glucose (RBG) ≥ 11.1 mmol/L (200 mg/dL) or blood glucose ≥ 11.1 mmol/L (200 mg/dL) 2 h after oral glucose tolerance test (OGTT) [1].

The increasing prevalence of diabetes mellitus is becoming a worldwide health problem. In 2017, it was reported that about 451 million people across the world had diabetes, with a projected increase to 693 million by 2045 [2]. As of 2021, the worldwide prevalence had increased to approximately 537 million worldwide, with projections of over 800 million cases by 2045. In the U.S., 1.2 million people are diagnosed with diabetes each year, and 40.1 million people are currently reported to be living with diabetes [3]. Several risk factors have been attributed to the increasing prevalence of diabetes, which include excessive smoking, poor nutrition, sedentary lifestyle, and circadian disruption.

Maintenance of normal circadian rhythms is crucial for sustaining a healthy life. Hence, aberrant light exposure, such as light at night, irregular work schedules, or frequent travel across time zones, may disrupt the circadian system, leading to desynchronization of circadian rhythm and increasing the risk for type 2 diabetes mellitus. In recent years, studies have revealed disruption of the circadian system as a predominant risk factor in the modern world. For instance, approximately 20% of the workforce is involved in shift work, while 33% of individuals report achieving less than 6 h of sleep [4], and up to 69% of people suffer jetlag [5]. Shift workers are at a greater risk of developing type 2 diabetes than day workers, with the highest risk reported among individuals working irregular rotating shifts that involve night shifts [6]. Notably, shiftwork alone was reported to increase the susceptibility of developing type 2 diabetes mellitus by 30% [7]. This review focuses on type 2 diabetes, which accounts for over 90% of diabetes cases worldwide and is closely linked to modifiable factors such as obesity, lifestyle, and circadian disruption, making it a critical target for mechanistic investigation and therapeutic intervention. We discuss recent advances in the circadian regulation of glucose metabolism and their implications for understanding the pathogenesis and treatment of type 2 diabetes; accordingly, the term “diabetes” in this review refers specifically to type 2 diabetes mellitus.

## 2. The Overview of the Circadian System

The circadian system is an endogenous time-keeping network that enables organisms to anticipate and adapt to predictable daily environmental changes, most notably the light–dark cycle. In mammals, circadian rhythms operate on an approximately 24 h cycle and regulate a wide range of physiological processes, including sleep–wake behavior, hormone secretion, energy metabolism, and glucose homeostasis [8–13]. These rhythms are generated at the cellular level by self-sustaining molecular clocks and are hierarchically organized to ensure temporal coordination across tissues.

In mammals, there is a hierarchy of circadian oscillators that control effective circadian timekeeping throughout the body. The human “master clock” resides in the hypothalamic suprachiasmatic nuclei (SCN) [14,15]. SCN neurons are critical for the generation of robust circadian rhythms and orchestrate rhythms in the vast majority of physiological and behavioral processes through neural and humoral output signals [16]. Light is the primary cue that entrains the SCN pacemaker to daily variations in the light/dark (LD) oscillations [17]. The SCN receives photic input from the retina and synchronizes internal circadian timing with the external environment. To synchronize the clock with the 24 h day, light travels to the SCN through the retinohypothalamic tract [18]. Melanopsin-sensitive retinal pathways transmit signals that enable the master clock to adjust to environmental light cycles [19]. Light-mediated circadian neural pathways are separate from the traditional image-forming vision pathway mediated by rods and cones [20]. Although the central hypothalamic clock coordinates all the body’s circadian rhythms, the majority of the body’s systems, organs and cells, including those vital for metabolic regulation like the adipose tissues, skeletal tissues and cells, renal tissues, hepatocytes, and some of the islet cells in the pancreas, possess their own autonomous circadian oscillators [21]. Importantly, these peripheral clocks can also be influenced by non-photic cues, including feeding–fasting cycles, physical activity, and hormonal signals, allowing metabolic tissues to respond dynamically to nutrient availability.

**Figure 1.**
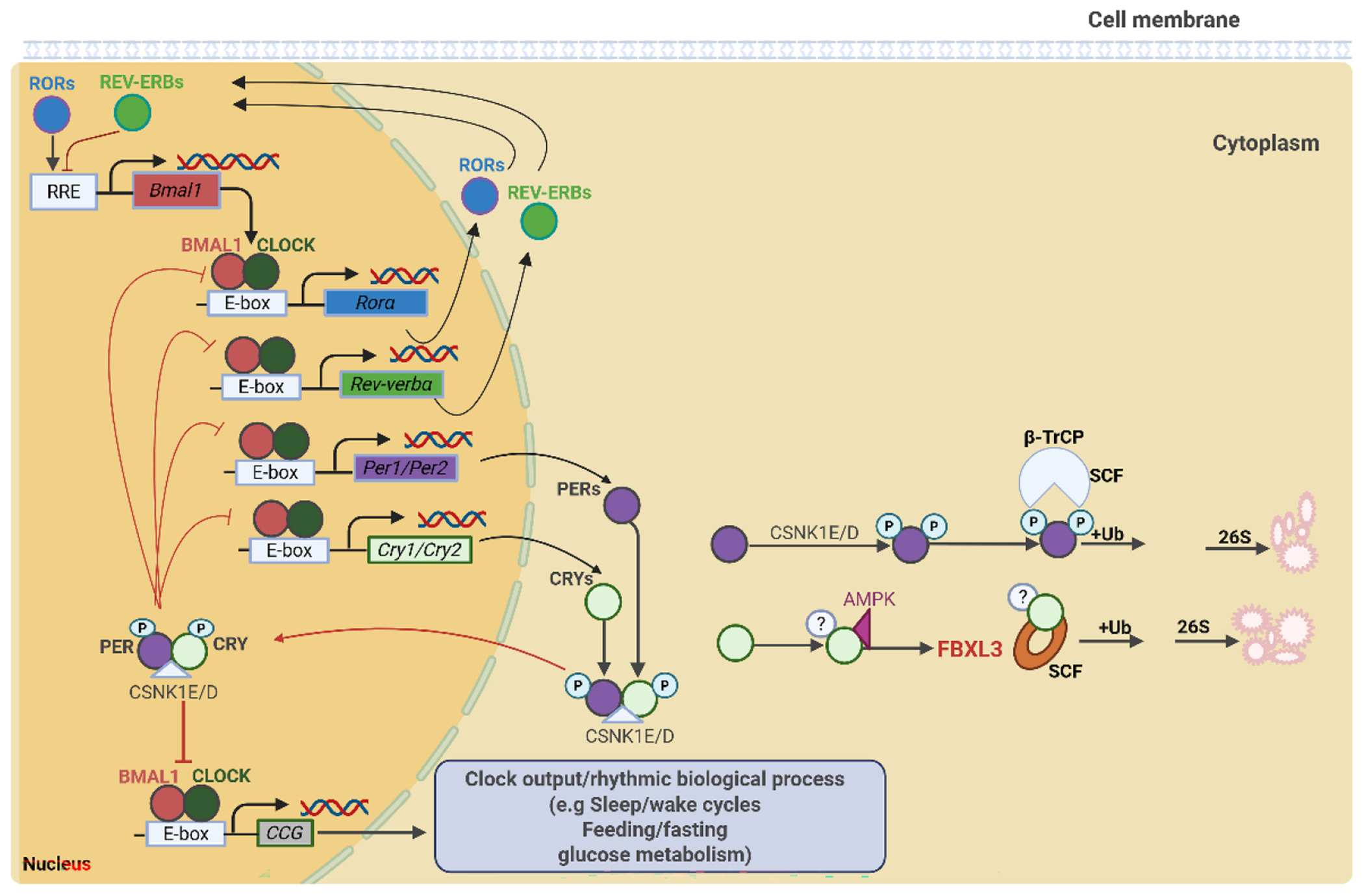
Model of the mammalian circadian clock. The SCN, the central clock in the hypothalamus, synchronizes peripheral clocks and regulates the body’s circadian system. The *CLOCK/BMAL1* heterodimer complex influences target gene E-box elements, initiating the transcription of core clock genes. *PER* and *CRY* proteins build up in the cytoplasm, forming heterodimers that move around the cytoplasm and nucleus. After phosphorylation by CK1δ and CK1ε, these complexes repress *CLOCK/BMAL1*-driven transcription, suppressing E-box gene expression. Additionally, *REV-ERBα* binds to the *ROR* response element (RRE) in the promoter region to repress Bmal1 expression, while *RORα* counteracts this effect by promoting its activation.

At the molecular level, circadian rhythms are driven by interlocking transcription–translation feedback loops [14,22]. The core clock components *CLOCK* and *BMAL1* form a heterodimer that activates the transcription of target genes, including the Period (*Per1–3*) and Cryptochrome (*Cry1–2*) families. Accumulation of *PER* and *CRY* proteins subsequently inhibits *CLOCK–BMAL1* activity, generating a rhythmic cycle of gene expression. Additional stabilizing loops involving nuclear receptors such as *REV-ERBs* and *RORs* fine-tune the amplitude and precision of the clock. This core clock complex regulates the rhythmic transcription of a broad array of clock-controlled genes, including those involved in metabolic regulation, through interconnected positive and negative transcriptional–translational feedback loops (Figure 1). Collectively, these molecular oscillations regulate the rhythmic expression of thousands of clock-controlled genes, many of which are directly involved in glucose and lipid metabolism. The tight coupling between the circadian system and metabolic pathways is particularly evident in glucose regulation. Key processes such as insulin secretion, insulin sensitivity, hepatic gluconeogenesis, and glucose uptake exhibit robust circadian variation. Disruption of circadian alignment, through shift work, irregular sleep patterns, or mistimed feeding, leads to desynchronization between central and peripheral clocks, resulting in impaired glucose tolerance and insulin resistance [23]. These observations highlight the circadian system as a critical regulator of metabolic health and provide a mechanistic framework linking circadian disruption to the pathogenesis of type 2 diabetes. Understanding the organization and function of the circadian system is therefore essential for elucidating how temporal misalignment contributes to metabolic disease. Moreover, it lays the foundation for chronotherapeutic strategies that align behavioral or pharmacological interventions with endogenous circadian rhythms to improve glycemic control in type 2 diabetes.

## 3. Circadian Regulation of Glucose Homeostasis in Different Tissues

**Figure 2.**
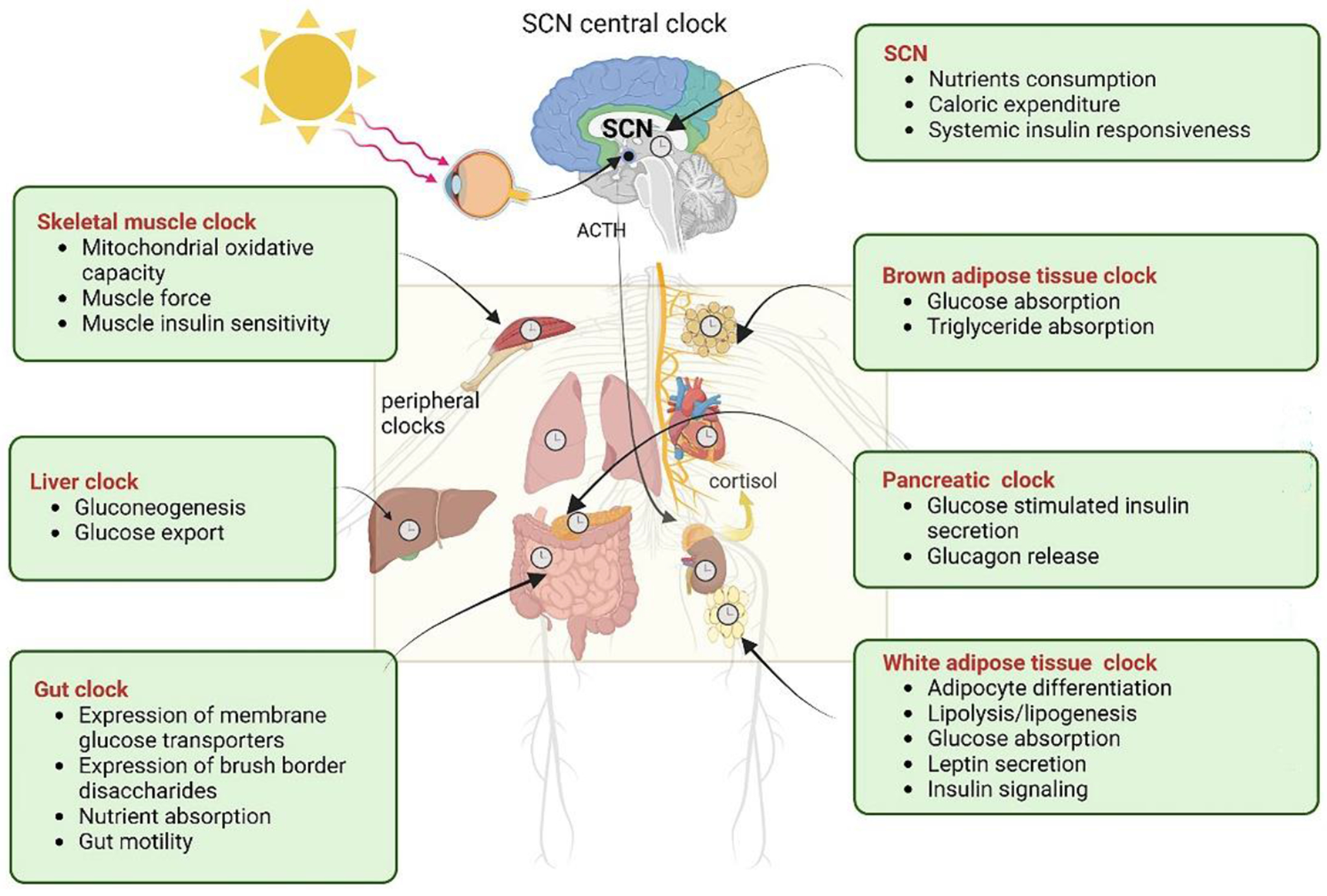
Central and peripheral clock regulation of glucose metabolism. The SCN functions as the central circadian pacemaker, but peripheral clocks in metabolic tissues such as the muscle, pancreas, liver, and adipose tissue can regulate glucose metabolism independently. These clocks autonomously control key processes, including gluconeogenesis, glycogen storage, and glucose release in the liver; insulin secretion in pancreatic β-cells; glucose uptake and insulin sensitivity in skeletal muscle; and lipolysis, glucose uptake, and adipokine secretion in adipose tissue. While the SCN synchronizes these clocks with external light-dark cycles, peripheral clocks are essential for maintaining rhythmic metabolic activity. However, misalignment between the SCN and peripheral clocks or disruptions within these autonomous clocks can contribute to insulin resistance, obesity, and diabetes, highlighting the vital role of circadian regulation in glucose homeostasis.

The central circadian clock, acting in synchrony with peripheral clocks, plays a critical role in regulating glucose metabolism. Figure 2 provides a schematic overview of how the suprachiasmatic nucleus coordinates tissue-specific peripheral clocks to control glucose production, uptake, storage, and utilization across metabolic organs. The circadian timing system plays a fundamental role in the regulation of glucose homeostasis, coordinating metabolic processes with predictable daily cycles of feeding, fasting, activity, and rest [24,25]. The SCN coordinates peripheral circadian oscillators through a combination of neural, hormonal, behavioral, and metabolic signals. Key mechanisms include autonomic nervous system output, rhythmic secretion of glucocorticoids and melatonin, feeding–fasting cycles, and daily fluctuations in body temperature. Together, these signals synchronize clock gene expression in peripheral metabolic tissues, thereby ensuring temporal alignment of physiological processes involved in glucose homeostasis and energy metabolism. In humans, circulating glucose levels exhibit a robust circadian rhythm, characterized by a rise in the early morning hours upon awakening, a phenomenon commonly referred to as the dawn phenomenon [26]. This rhythmic increase in blood glucose is thought to arise, at least in part, from circadian variations in counter-regulatory hormones that antagonize insulin action. Growth hormone secretion peaks during the night, followed by an early-morning surge in cortisol, both of which promote hepatic glucose production and transiently reduce insulin sensitivity [27–29].

In metabolically healthy individuals, functional pancreatic β-cells respond appropriately to this early-morning rise in glucose by increasing insulin secretion, thereby restoring normoglycemia. In contrast, in individuals with diabetes, where insulin secretion is impaired or insulin sensitivity is reduced, the dawn phenomenon becomes exaggerated and prolonged, contributing to sustained hyperglycemia and increased risk of long-term metabolic complications. While nutrient intake is a primary stimulus for insulin release, accumulating evidence indicates that insulin secretion and action are also strongly modulated by time of day, independent of food availability, as demonstrated in both human and animal studies [30–33].

Rodent models further support a circadian regulation of glucose metabolism, as fasting blood glucose levels display intrinsic daily oscillations even when feeding behavior is tightly controlled [34]. For instance, C57BL/6J mice maintained under standard light–dark conditions exhibit elevated fasting glucose levels at the onset of the active (early dark) phase and lower levels at the beginning of the rest (light) phase, a pattern analogous to the human dawn phenomenon [35]. These observations underscore the importance of circadian rhythms in aligning glucose production, utilization, and storage with behavioral and environmental cycles. Collectively, these findings highlight the necessity of an intact circadian system for normal glucose regulation and metabolic flexibility. Proper coordination between the central clock and peripheral clocks within metabolic tissues, including the pancreas, liver, skeletal muscle, adipose tissue, and gut, is essential for maintaining daily glucose balance. Disruption of circadian organization, as increasingly observed in modern lifestyles characterized by shift work, jet lag, irregular sleep patterns, and excessive exposure to artificial light, can profoundly interfere with this coordination and predispose individuals to metabolic disorders such as obesity and type 2 diabetes mellitus.

Recent research has increasingly focused on understanding how the circadian clock genes coordinate metabolic processes at both central and peripheral levels. The functional roles of these core clock components have been extensively investigated across multiple metabolic organs and tissues, where they have been shown to exert critical control over glucose metabolism. Key findings from these studies are summarized in Table 1. The following sections examine in detail how circadian regulation of glucose homeostasis is implemented within individual metabolic tissues and how its disruption contributes to metabolic disease.

### 3.1. Pancreatic Islets

Pancreatic islets play a central role in circadian regulation of glucose homeostasis by coordinating insulin and glucagon secretion with daily rhythms in feeding and activity. Early physiological studies demonstrated that glucose tolerance and insulin secretory responses exhibit pronounced diurnal variation, with enhanced insulin responsiveness occurring during the early active phase, coinciding with peak food intake [31,54]. Subsequent investigations in metabolically healthy humans revealed robust circadian rhythms in insulin secretion that persist independently of feeding behavior and circulating glucose levels, indicating intrinsic time-of-day regulation of β-cell function [55]. These observations are supported by in vitro studies showing circadian rhythmicity in glucose-stimulated insulin secretion in isolated pancreatic β-cells, highlighting the importance of a cell-autonomous circadian clock in regulating islet hormone output [56,57]. Collectively, these findings establish circadian control of pancreatic hormone secretion as a fundamental determinant of daily glucose homeostasis.

Circadian regulation within the pancreas is mediated by intrinsic molecular clocks that operate autonomously within islet cells. Although the circadian modulation of α-cell-derived glucagon secretion remains less well characterized, available in vivo and in vitro evidence demonstrates rhythmic oscillations in glucagon production, suggesting coordinated temporal regulation of both insulin and glucagon release [57,58]. These findings prompted the development of in vitro reporter models using clock gene-driven luciferase to monitor circadian oscillations in pancreatic islets. Using these approaches, multiple studies have demonstrated the presence of a self-sustained circadian clock within β-cells, characterized by rhythmic expression of core clock genes with defined amplitudes and approximately 24 h periodicity [37,59–63]. Importantly, these data confirm that pancreatic islets possess an intrinsic circadian timing system capable of regulating endocrine function independently of external cues.

The synchronization and integrity of the pancreatic islet circadian clock are influenced by central circadian signals and environmental lighting conditions. Evidence indicates that the suprachiasmatic nucleus (SCN) indirectly modulates islet rhythmicity through its regulation of feeding behavior, thereby contributing to the entrainment of peripheral clocks within the pancreas. Experimental disruption of normal light–dark cycles, particularly prolonged light exposure, has been shown to impair islet circadian function in rodent models, resulting in β-cell apoptosis, disrupted insulin rhythmicity, and hyperglycemia, effects that are further exacerbated under high-fat diet conditions [60,61]. These findings underscore the vulnerability of pancreatic islet clocks to circadian misalignment and highlight their contribution to metabolic dysfunction under conditions of environmental circadian disruption.

At the molecular level, circadian clocks directly regulate genes critical for β-cell metabolism, insulin secretion, and survival [62,63]. Transcriptomic analyses have revealed robust circadian oscillations in genes involved in β-cell function, with distinct temporal peaks and troughs across the day [64,65]. Notably, the aryl hydrocarbon receptor nuclear translocator (*Arnt*), a key regulator of β-cell metabolic capacity, is rhythmically upregulated by the clock-controlled transcription factor D-box binding protein (*Dbp*), reaching peak expression at Zeitgeber time 4 in mouse models [65]. In parallel, circadian modulation of β-cell potassium ion conductance closely aligns with rhythmic insulin secretion, reflecting clock-dependent control of membrane excitability. Core clock components, including CLOCK and REV-ERBα, further regulate insulin exocytosis by controlling the expression of *syntaxin-1* and *SNAP25*, while also influencing β-cell development, survival, and proliferation through key regulatory pathways involving *InsR* and *Pdx1* [64]. Together, these molecular mechanisms position the pancreatic islet circadian clock as a critical regulator of β-cell function and glucose homeostasis.

### 3.2. Skeletal Muscles

Skeletal muscle possesses a robust and autonomous circadian clock that plays a central role in the regulation of glucose metabolism. Circadian oscillators have been identified in myocytes both in vitro and in vivo [66–68], and nearly all skeletal muscle processes essential for glucose homeostasis display marked diurnal rhythmicity. These include glucose uptake and oxidation via the GLUT4 transporter [38], glycogen synthesis and storage [69,70], mitochondrial oxidative metabolism [71], and protein and lipid turnover [72,73]. Consistent with these rhythms, studies in both rodents and humans demonstrate that glucose uptake by skeletal muscle peaks at the onset of the active phase, aligning energy utilization with behavioral demands. Notably, isolated myocytes retain rhythmic patterns of glucose uptake ex vivo, indicating that these processes are governed by a self-sustained peripheral clock independent of systemic cues [66]. Together, these findings establish skeletal muscle as a clock-regulated metabolic tissue optimized for the temporal coordination of glucose utilization. Disruption of the skeletal muscle circadian clock has been strongly implicated in the development of metabolic dysfunction and diabetes. Early investigations focused on the core clock gene *Bmal1* (also known as MOP3), which is indispensable for circadian rhythmicity [74]. Global deletion of *Bmal1* in mice results in rapid loss of circadian rhythms under constant darkness and reduced physical activity even under normal light–dark cycles, underscoring its fundamental role in clock function. Subsequent studies confirmed that *Bmal1* is essential for maintaining biological rhythms and demonstrated that skeletal muscle physiological deficits observed in global *Bmal1* knockout mice could be rescued by restoring *Bmal1* expression specifically in muscle tissue [75,76]. These observations highlight the importance of an intact muscle clock in sustaining normal metabolic physiology.

Muscle-specific deletion of *Bmal1* has provided direct insight into how the skeletal muscle clock controls glucose metabolism. Mice lacking *Bmal1* selectively in skeletal muscle, whether during development or adulthood, exhibit pronounced glucose intolerance and insulin resistance driven primarily by impaired insulin-stimulated glucose uptake [39,77]. This phenotype closely resembles the reduced insulin sensitivity characteristic of type 2 diabetes. Mechanistically, *Bmal1* deficiency leads to reduced expression of TBC1D1, a Rab-GTPase critical for GLUT4 translocation, as well as decreased protein levels of GLUT4 itself, resulting in blunted glucose transport into myocytes [39]. These findings demonstrate that the skeletal muscle clock is essential for maintaining insulin responsiveness and efficient glucose disposal. In addition to regulating glucose transport, the skeletal muscle clock orchestrates intracellular glucose oxidation and metabolic flexibility. Muscle-specific *Bmal1* knockout mice display altered expression of clock-controlled genes such as *Pdk4* and *Pdp1*, which encode pyruvate dehydrogenase (PDH) kinase and phosphatase, respectively, leading to reduced PDH activity and impaired glucose oxidation. Furthermore, enzymes critical for glucose utilization, including hexokinase and PDH, are reduced in *Bmal1*-deficient muscle, promoting a metabolic shift toward increased lipid utilization and storage. Importantly, skeletal muscle from individuals with diabetes exhibits similar defects in glucose transport and metabolism [78], reinforcing the translational relevance of these findings. Collectively, these data demonstrate that the intrinsic skeletal muscle clock is indispensable for coordinating glucose uptake, oxidation, and storage across the rest–activity cycle, and that its disruption contributes directly to the pathogenesis of insulin resistance and diabetes.

### 3.3. Hepatocytes

The liver serves as a central metabolic organ that integrates circadian timing cues to maintain daily glucose homeostasis. As the primary organ responsible for endogenous glucose production, the liver determines fasting and post-absorptive blood glucose levels through tightly regulated circadian oscillations in hepatic glucose output [21]. During fasting, temporal variations in circulating glucose are largely driven by rhythmic changes in gluconeogenesis and glycogenolysis, processes that are coordinated by both the central circadian pacemaker and the intrinsic hepatic clock.

Disruption of either the master clock in the suprachiasmatic nucleus or the liver-specific circadian clock leads to loss of diurnal glucose rhythms and impaired glycemic control, underscoring the liver’s indispensable role in circadian regulation of systemic glucose metabolism [15,70,79]. Circadian control of hepatic glucose production is closely linked to temporal variation in insulin sensitivity and postprandial glucose handling. Rhythmic gluconeogenesis not only shapes fasting glucose levels but also influences time-of-day-dependent insulin responsiveness, thereby modulating postprandial glucose excursions [55,58]. Both human and rodent studies have demonstrated that major physiological drivers of hepatic glucose production, including hormonal signaling, substrate availability, and enzymatic activity, are under circadian regulation [70,80,81]. Consequently, misalignment between circadian timing and nutrient intake, as observed in shift work or irregular feeding schedules, disrupts hepatic glucose regulation and promotes metabolic dysfunction. Thus, circadian regulation of hepatocyte function is critical for synchronizing hepatic glucose output with systemic insulin action across the daily cycle.

In addition to canonical gluconeogenic pathways, circadian regulation of hepatic glucose metabolism involves lipid–glucose crosstalk mediated by clock-controlled metabolic sensors. Fatty acid translocase CD36, a scavenger receptor abundantly expressed in hepatocytes, has emerged as a key integrator of lipid handling and glucose homeostasis [82]. Clinical studies reveal that individuals with inherited CD36 deficiency exhibit increased susceptibility to insulin resistance and glucose intolerance [83–85], highlighting its metabolic relevance. More recent work using liver-specific CD36 knockout models demonstrates that CD36 expression itself is rhythmic and that its absence disrupts hepatic clock function and destabilizes daily glucose rhythms [86]. These findings identify CD36 as a critical clock-regulated mediator linking hepatic lipid flux to circadian glucose regulation. Beyond metabolic flux, hepatocytes exhibit intrinsic circadian rhythms at the transcriptional, translational, and structural levels. Genome-wide analyses have revealed robust oscillations in hepatic gene expression and protein abundance, accompanied by rhythmic changes in hepatocyte size, volume, and cellular architecture [87–89]. These structural and molecular rhythms reflect the coordinated temporal regulation of liver function, optimizing metabolic capacity according to the energetic demands of the rest–activity cycle. Importantly, these intrinsic rhythms persist even in isolated hepatocytes, supporting the presence of a self-sustained peripheral clock within liver cells. Together, these observations demonstrate that circadian regulation of hepatocyte function extends beyond metabolism to encompass fundamental aspects of cellular organization and physiology.

At the molecular level, the hepatic circadian clock orchestrates daily rhythms in genes governing glucose production, uptake, storage, and lipid metabolism. Key gluconeogenic and glycogenolytic genes, including *Fbp1*, *Pcx*, *G6Pase*, *Pepck*, *Pyg*, and the transcription factor *FoxO1*, exhibit pronounced circadian oscillations that regulate glucose output [90–93]. Similarly, genes involved in hepatic glucose uptake and storage—such as *Slc2a2* (GLUT2), *Gck*, *Hk2*, *Pklr*, and *G6pdx*—are rhythmically expressed, ensuring efficient glucose clearance during feeding periods. In parallel, clock-controlled lipid metabolism genes (*Slc27a1*, *Acaca*, *Cpt1a*, *Lipin1*, *Pparα*, and *Dgat1*) coordinate lipid oxidation and storage, thereby influencing hepatic insulin sensitivity and substrate selection. This integrated transcriptional program highlights how the hepatic clock synchronizes glucose and lipid metabolism to maintain metabolic flexibility.

Importantly, hepatic circadian rhythms are shaped by both intrinsic clock mechanisms and extrinsic cues, particularly feeding–fasting cycles. While the hepatocyte clock drives cell-autonomous rhythmicity, feeding time acts as a dominant zeitgeber that can reinforce or override endogenous oscillations [89]. As a result, some hepatic rhythms are strictly clock-dependent, whereas others are primarily driven by nutrient availability and hormonal signaling. Disentangling these clock-dependent and clock-independent components is essential for accurately interpreting circadian regulation in the liver, especially in experimental models involving restricted feeding or circadian disruption. Therefore, hepatic glucose rhythms arise from a dynamic interplay between intrinsic molecular clocks and behavioral timing cues. In general, the hepatic circadian clock plays a pivotal role in regulating daily glucose metabolism by coordinating glucose production, uptake, storage, and substrate utilization in alignment with systemic physiological rhythms. Through tight temporal control of gluconeogenesis, glycogen metabolism, and lipid–glucose integration, hepatocytes ensure metabolic homeostasis across fasting and feeding states. Disruption of hepatic circadian timing, whether through genetic manipulation, altered feeding patterns, or environmental circadian misalignment, leads to impaired glycemic control and increased susceptibility to metabolic disease. These findings underscore the liver as a critical circadian metabolic organ and highlight circadian regulation as a fundamental determinant of glucose homeostasis and metabolic health.

### 3.4. Adipocytes

Adipose tissue is a metabolically active organ that plays a central role in the circadian regulation of systemic glucose homeostasis. The intrinsic circadian clock within adipocytes governs key metabolic processes, including insulin sensitivity, glucose uptake, lipid storage, and adipokine secretion, all of which are essential for maintaining metabolic balance. Disruption of adipocyte circadian rhythms has been strongly associated with obesity, insulin resistance, and the development of type 2 diabetes. White adipose tissue (WAT), in particular, functions not only as the body’s primary energy reservoir but also as a critical endocrine organ that modulates whole-body glucose metabolism through dynamic lipid handling and hormone secretion. Thus, circadian regulation of adipocyte function is fundamental to systemic metabolic homeostasis. Circadian control of lipid storage and mobilization in adipose tissue is essential for maintaining glucose homeostasis. WAT stores excess energy in the form of triglycerides, while regulated lipolysis ensures appropriate release of free fatty acids (FFAs) during fasting or increased energy demand. Dysregulated lipolysis results in elevated circulating FFAs, which impair insulin signaling in peripheral tissues and promote insulin resistance, a hallmark of diabetes [94]. Plasma FFA levels exhibit robust circadian rhythmicity, reflecting time-of-day-dependent regulation of adipose tissue insulin signaling and lipid metabolic pathways [95]. Consequently, precise circadian coordination of lipogenesis and lipolysis in adipocytes is required to prevent lipotoxicity and preserve insulin sensitivity. Mounting evidence demonstrates that key adipose tissue functions are under strong circadian regulation in both humans and rodent models. Physiological processes such as lipolysis, adipogenesis, insulin responsiveness, and adipose tissue inflammation display pronounced daily oscillations driven by the adipocyte clock [96–98].

Genetic disruption of core clock components further underscores this relationship, as *Clock* and *Bmal1* mutant mice consistently develop obesity, hyperlipidemia, and elevated circulating FFAs and triglycerides [99,100]. These phenotypes highlight the importance of intact circadian timing in adipose tissue for maintaining lipid and glucose balance. Collectively, these findings establish adipocyte circadian rhythms as critical determinants of metabolic health.

At the molecular level, adipocytes exhibit rhythmic expression of numerous clock-controlled genes that regulate lipid and glucose metabolism. Genome-wide analyses in both human and mouse adipose tissue have revealed robust circadian oscillations in genes involved in lipid synthesis, breakdown, and energy expenditure [96,101,102]. Core clock components such as *Bmal1* and *Clock* directly regulate lipolytic activity and lipid energy dissipation, particularly during the light (rest) phase [103]. Disruption of *Bmal1* in adipocytes leads to impaired synthesis of polyunsaturated fatty acids, driven by reduced expression of *Scd1* and *Elovl6*, enzymes critical for lipid desaturation and elongation. These molecular defects further link adipocyte clock dysfunction to altered lipid composition and metabolic imbalance. Beyond its role in energy storage, adipose tissue functions as one of the largest endocrine organs, secreting adipokines that influence systemic insulin sensitivity and glucose metabolism. Hormones such as leptin, adiponectin, and pro-inflammatory cytokines exhibit circadian patterns of expression and release, contributing to daily fluctuations in glucose tolerance and insulin action [104]. Circadian disruption in adipose tissue alters adipokine profiles, promoting chronic low-grade inflammation and insulin resistance—key drivers of metabolic disease. Therefore, circadian regulation of adipokine secretion represents a critical mechanism by which adipose tissue communicates temporal metabolic information to other organs. In summary, the adipocyte circadian clock plays an essential role in regulating glucose metabolism by coordinating lipid storage, mobilization, insulin sensitivity, and endocrine signaling in a time-of-day-dependent manner. Synchronization of the adipose clock with other peripheral metabolic clocks ensures balanced energy distribution and metabolic flexibility across feeding–fasting cycles. Conversely, disruption of adipocyte circadian rhythms contributes to obesity, insulin resistance, and diabetes, highlighting adipose tissue as a key circadian regulator of metabolic health. Together, these findings underscore the importance of circadian timing in adipose tissue as a fundamental determinant of systemic glucose homeostasis.

## 4. Circadian Rhythm and Diabetes Mellitus

The circadian timing system plays an essential role in maintaining metabolic homeostasis, particularly in the regulation of glucose metabolism and insulin signaling. Under normal physiological conditions, several metabolic processes exhibit clear circadian patterns, including glucose tolerance, insulin sensitivity, and pancreatic insulin secretion. These temporal variations allow the body to efficiently manage nutrient intake and energy utilization during active and resting phases. Disruption of these rhythmic processes can impair metabolic regulation and contribute to the development of metabolic disorders. Diabetes mellitus is a chronic metabolic disease characterized by persistent hyperglycemia resulting from impaired insulin secretion, insulin resistance, or both. Increasing evidence suggests that disturbances in circadian rhythms are closely associated with the onset and progression of diabetes. Under normal physiological conditions, increased insulin production and secretion by pancreatic β-cells promote the absorption of blood glucose into peripheral tissues and suppress gluconeogenesis in the liver. To maintain blood glucose balance, insulin binds to receptors in various cells in the muscles, hepatocytes, and adipocytes, thereby enhancing glucose uptake into these tissues [105]. Once absorbed, glucose is either converted into energy, stored as triglycerides in adipose tissue, muscle, and liver, or stored as glycogen, which acts as a reserve to stabilize blood glucose levels [106]. Diabetes is driven by two main pathophysiological factors, namely, insulin resistance in insulin-sensitive tissues and impaired pancreatic β-cell function. Persistent hyperglycemia exacerbates insulin resistance in peripheral tissues, diminishing their sensitivity to insulin. Pancreatic β-cells secrete more insulin during the prediabetic stage to counteract insulin resistance and maintain normoglycemia. Chronically high insulin secretion, on the other hand, causes an eventual insulin shortage due to impaired pancreatic β-cell functions, which in turn redevelops hyperglycemia and eventually diabetes [107]. Importantly, emerging evidence indicates that disturbances in circadian regulation and cellular redox balance play critical roles in the initiation and progression of these pathophysiological processes. Circadian misalignment alters insulin sensitivity, β-cell function, and hepatic glucose production, while oxidative stress exacerbates insulin resistance and promotes β-cell dysfunction. These mechanisms act both independently and synergistically to accelerate metabolic deterioration and disease progression. Accordingly, understanding how circadian rhythm disruption and oxidative stress contribute to diabetes pathogenesis is essential for identifying novel therapeutic strategies and will be discussed in detail in the following sections.

### 4.1. Circadian Disruption and Pathogenesis of Diabetes Mellitus

Circadian disruption, arising from a variety of environmental and behavioral factors, exerts deleterious effects on blood glucose regulation and accelerates the onset and progression of diabetes. One of the most important mechanisms controlling glucose metabolism is the circadian clock. Physiologically, the SCN, in synchrony with other clocks found in the metabolic tissues, ensures adequate 24 h glucose control. This is achieved by coordinating physiological and biochemical events that are associated with glucose metabolism such as hormonal secretion, insulin sensitivity, absorption of glucose and metabolic enzyme peaks and troughs. The diurnal oscillations of the enzymes responsible for glycolysis, gluconeogenesis, and glycogen synthesis guarantee that glucose production and use correspond optimally with energy demands. In individuals who are exposed to shift work, insulin sensitivity is greatly altered, resembling impairments seen in type 2 diabetes mellitus. In a similar manner, these subjects were shown to express higher inflammatory markers that are associated with insulin resistance [108]. Moreso, circadian misalignment among shift workers presented with impaired glucose tolerance [109]. More compelling evidence came from a recent study in a large cohort of subjects (670,000), where individuals who were exposed to brighter night light and light patterns that can interfere with circadian rhythms had a greater chance of developing diabetes [110]. Furthermore, compared to those who achieve the recommended 7–8 h of sleep per night, people with both short and extended sleep durations are more likely to experience the advancement of diabetes [111]. Irregular or uncontrolled exposure to natural light over a prolonged period has been shown to contribute to the advancement of key metabolic malfunctions, particularly diabetes.

To further understand this concept, the effect of circadian disruption using aberrant light exposure in C57/BL/6J mice was recently studied by exposing mice to 8h/8h light/dark cycle from the start of gestation and continuing through adulthood. Findings show that circadian disruption during developmental stages and across lifespan increases predisposition to adult metabolic disorders [112]. It was shown that adult mice displayed impaired glucose and insulin sensitivity in a manner similar to type 2 diabetes. Mechanistically, these metabolic abnormalities were associated with defective insulin signaling in both hepatic and skeletal muscle tissues, highlighting peripheral insulin resistance as a major contributor to the observed phenotype. Collectively, these findings provide compelling experimental evidence that developmental circadian disruption induced by aberrant light exposure programs long-term metabolic dysfunction and establishes a direct causal link between circadian misalignment and impaired glucose metabolism [112]. The circadian system of the body is instrumental in the occurrence and regulation of various bodily functions in tandem and within a 24 h period. However, exposure to irregular light patterns at abnormal hours exposes an individual to altered circadian rhythms, leading to impairment of glucose metabolism and insulin action. Furthermore, misalignment in diurnal activities alters the normal functioning of β-cells; hence, insulin production and secretion are decreased while insulin sensitivity is also compromised, and all these lead to the development of diabetes. Additionally, disturbances in light-dark patterns alter metabolic pathways related to lipid and glucose metabolism, increasing glucose intolerance along with susceptibility to diabetes. These findings emphasize the role of circadian rhythm in metabolism and suggest that regulating abnormal exposure to environmental light may be considered as curative measures or preventive interventions for diabetes mellitus.

Further evidence linking circadian disruption to diabetes etiology comes from studies using animal models with targeted mutations in core circadian clock genes, which consistently demonstrate profound impairments in glucose homeostasis and insulin sensitivity. Mice carrying mutations in core clock genes, including *Bmal1* or *Clock*, have increased insulin resistance, poor glucose tolerance, high insulin levels, and a higher risk of obesity and diabetes. Furthermore, these mutant mice show reduced expression of genes related to islet growth and maturation, glucose tolerance, and insulin signaling [37]. Human studies support the connection between diabetes and gene variations associated with the circadian clock. Yamaguchi and colleagues found a significant correlation between variations in the *BMAL2* gene and the prevalence of diabetes in the Japanese population through genome-wide association studies [113]. Similarly, earlier research reported a strong link between specific *BMAL1* haplotypes and an increased risk of diabetes in the British population [114], while variations in the *CLOCK* gene also elevate the risk of developing diabetes [115]. These findings underscore the essential role of circadian rhythms in regulating metabolism and maintaining glucose balance. Consequently, disturbances in the circadian clock significantly influence the molecular mechanisms that drive the etiology and progression of diabetes.

### 4.2. Circadian Regulation of Molecular Pathways Associated with Pathogenesis of Diabetes

The onset and progression of diabetes are caused by complex biological pathways that disturb glucose homeostasis as part of the disease’s etiology. These molecular pathways are greatly influenced by the circadian clock and include insulin signaling, oxidative stress, endoplasmic reticulum (ER) stress, and mitochondrial malfunction, among other pathways. Here, we highlight some important circadian-associated molecular pathways involved in the pathogenesis and pathophysiology of type 2 diabetes mellitus.

#### 4.2.1. Role of mTOR in Glucose Homeostasis

**Figure 3.**
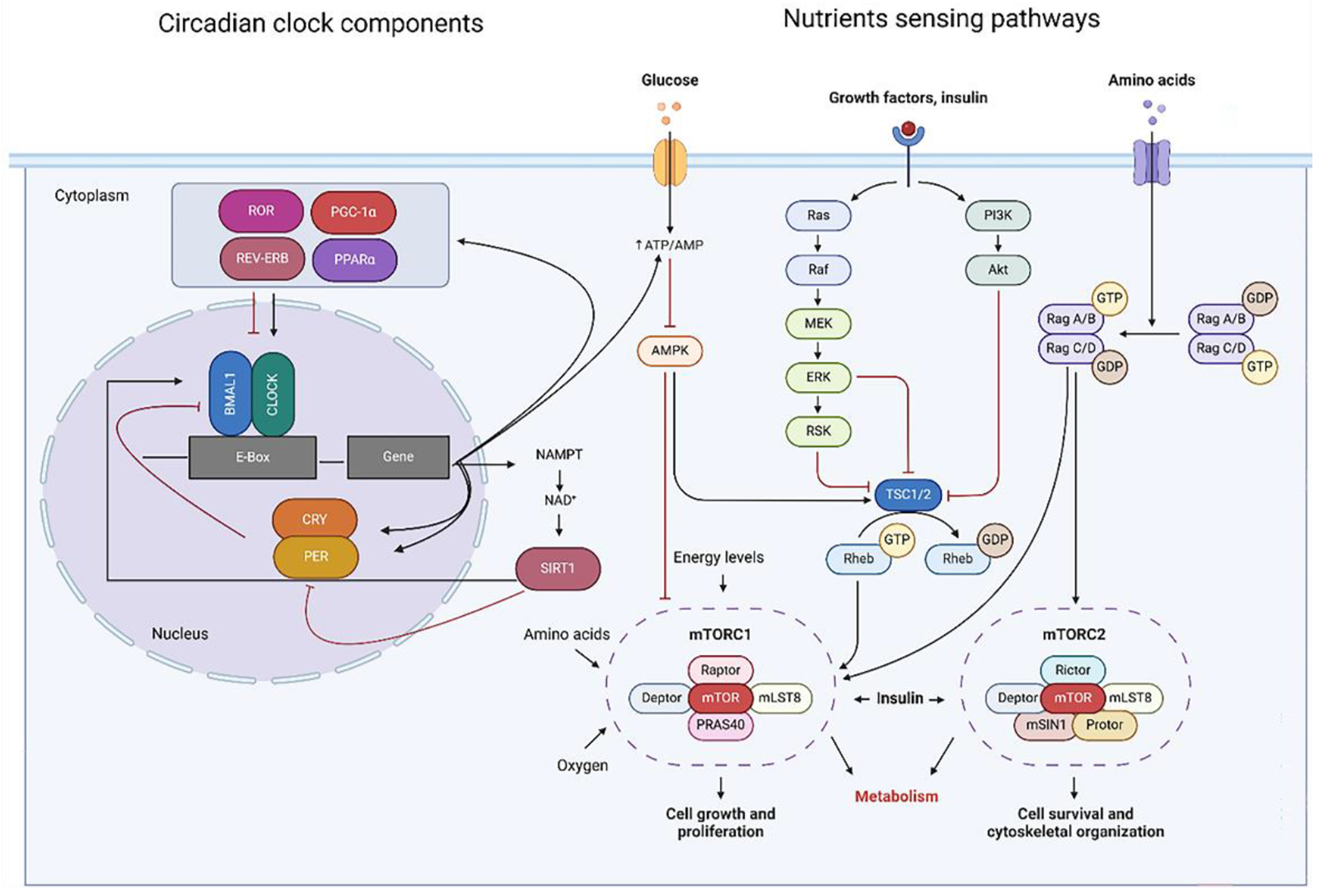
Circadian clock regulates mTOR activity in glucose homeostasis. Clock genes regulate mTOR activity by modulating the cellular energy state in synchrony with the circadian cycle, primarily through the glucose-ATP/AMP pathway. During the active phase, when glucose levels and ATP are high, genes like *BMAL1* and *CLOCK* promote mTOR activation by inhibiting AMP-activated protein kinase (AMPK), which normally suppresses mTOR when ATP is low and AMP is elevated. This high ATP/AMP ratio, driven by clock-controlled metabolic processes, supports protein synthesis, cell growth, and metabolism by favoring mTOR activation. Conversely, during the rest phase, when glucose is low and AMP is high, clock genes promote AMPK activation, inhibiting mTOR and conserving energy while suppressing anabolic processes. The mTOR signaling pathway is critical for the growth and differentiation of many cells and serves to regulate the state of energy balance within the organism. Two important complexes, mTORC1 and mTORC2, are responsible for maintaining glucose balances, lipid levels, and insulin action on the AKT pathway, respectively, with an aim to maintain blood glucose. In response to nutrients, such as glucose and amino acids, the insulin signaling pathway is triggered and causes AKT (Protein Kinase B) activation. This is followed by a series of other steps where AKT initiates the inhibition of TSC1/2 complex to enhance Rheb, a GTPase whose function is direct stimulation of mTORC1, resulting in its activation. The Rag GTPase also helps in the translocation of mTORC1, where it is triggered by Rheb and leucine on the lysosomal surface. Once the mTORC1 is activated, it activates proteins such as the S6K1 and 4E-BP1, and is also involved in the control of fat metabolism and autophagy. mTORC2, in this way, is important to the overall balance of signals as it modulates the activity of AKT and cell survival pathways. Such pathways and particularly, chronic overload of nutrition could lead to constant activation of mTORC1 and development of complications such as insulin resistance, impaired glucose uptake, hence exposing the beta cells to oxidative stress, a key player that worsens diabetes.

The mammalian/mechanistic target of rapamycin (mTOR) is an essential serine/threonine (Ser/Thr) kinase that regulates vast physiological and biochemical processes, some of which include cell growth, development, proliferation and survival, autophagy and neuroendocrine metabolism [116]. There are two distinct functional complexes of mTOR characterized by their unique components and the biological processes they mediate. mTOR complex1 (mTORC1) is characterized by the presence of regulatory-associated protein of mammalian target of rapamycin (RAPTOR), while mTOR complex2 (mTORC2) comprises rapamycin-insensitive companion of mTOR (RICTOR), which regulates their respective substrate binding abilities. Circadian clocks play a major role in regulating the daily rhythms of the mTOR pathway to modulate the rate of protein synthesis to match the body’s metabolic state and energy needs (Figure 3). Disturbances in the circadian regulation of mTOR signaling in diabetes may intensify metabolic dysregulation, culminating in β-cell malfunction and reduced insulin response [117]. This suggests that the pathophysiology of diabetes mellitus is heavily dependent on mTOR. Overactivation of mTORC1 exacerbates the metabolic abnormalities associated with diabetes by impairing insulin signaling and encouraging fat buildup, inflammation, and oxidative stress. This can be explained in part by its role in phosphorylating downstream targets like S6 kinase and 4E-BP1. The desynchronization of glucose metabolism, fat buildup, and inflammatory responses can be caused by aberrant mTOR activity, namely mTORC1. This underscores the critical role that mTOR activity plays in the interplay between circadian rhythms and the pathophysiology of diabetes.

#### 4.2.2. Role of Integrated Stress Response in Glucose Homeostasis

Integrated stress response (ISR) is primarily a defense mechanism tightly controlled by circadian rhythms, with a crucial role in the modification of protein synthesis in response to cellular stimuli. Disruption in circadian rhythm in a diabetic state causes activation of the ISR, thereby resulting in prolonged activation of eukaryotic initiation factor 2 alpha (eIF2α) [118]. This chronic reaction alters the temporal control of protein synthesis and worsens endogenous stress to the endoplasmic reticulum and ultimately disrupts insulin synthesis and release. Some of the cellular stressors implicated in activating ISR in diabetes include diurnal timing in calorie intake and oxidative damage, which has also been shown to have a profound effect on the circadian clock [118,119]. Hence, understanding the circadian control of ISR in the pancreas and its corresponding effects on insulin sensitivity explains the need for maintaining an adequate internal clock system with the goal of ameliorating stress and producing optimal metabolic balance, especially in patients with type 2 diabetes mellitus.

#### 4.2.3. Role of MAPK, MNK and elF4E in Glucose Homeostasis

For proper synchronization of cellular responses with environmental cues, the mitogen-activated protein kinases (MAPKs) play crucial roles as transducers of extracellular signals. These crucial roles, which are mediated by the circadian clock, include regulation of insulin signaling and metabolism via the *ERK*, *JNK* and *p38* cascades [120]. Circadian disturbance in diabetes can result in impaired MAPK activity, which significantly impacts metabolic gene expressions, leading to apoptosis and insulin resistance [121]. It has been shown that the normal endogenous rhythmicity of insulin receptor substrate-1 (IRS-1) is affected because of JNK activation impairment. In a similar manner, desynchronization of *p38 MAPK* oscillations is implicated in the exacerbation of inflammatory responses and β-cell death. These may, in part, explain the involvement of circadian rhythm via the *MAPK* pathway in the pathogenesis of diabetes. Moreso, other important modulators of mRNA translation such as *MAPK*-interacting kinases (*MNKs*) and eukaryotic initiation factor 4E (*eIF4E*) have been found useful in understanding the pathogenesis of diabetes. Interestingly, these regulators are also shown to be under the influence of the circadian clock. Phosphorylation of *eIF4E* through the actions of *MNKs* increases its potential for mRNA translation, protein synthesis, cell development and survival, with crucial roles in insulin signaling [122]. Circadian disruption that occurs in diabetes results in misalignment of *MNK* oscillations and, in turn, disrupts the circadian rhythmicity of *elF4E*. This results in rapid translation of stress-associated proteins and proinflammatory cytokines, consequently, reducing insulin sensitivity and enhancing β-cell dysfunction. Targeting the circadian regulation of *MNK* and *eIF4E* activities as a treatment method has the potential to address their key involvement in the etiology of diabetes, as evidenced by the disruption of their control over these activities.

### 4.3. Circadian Rhythm of Oxidative Stress and Diabetes

**Figure 4.**
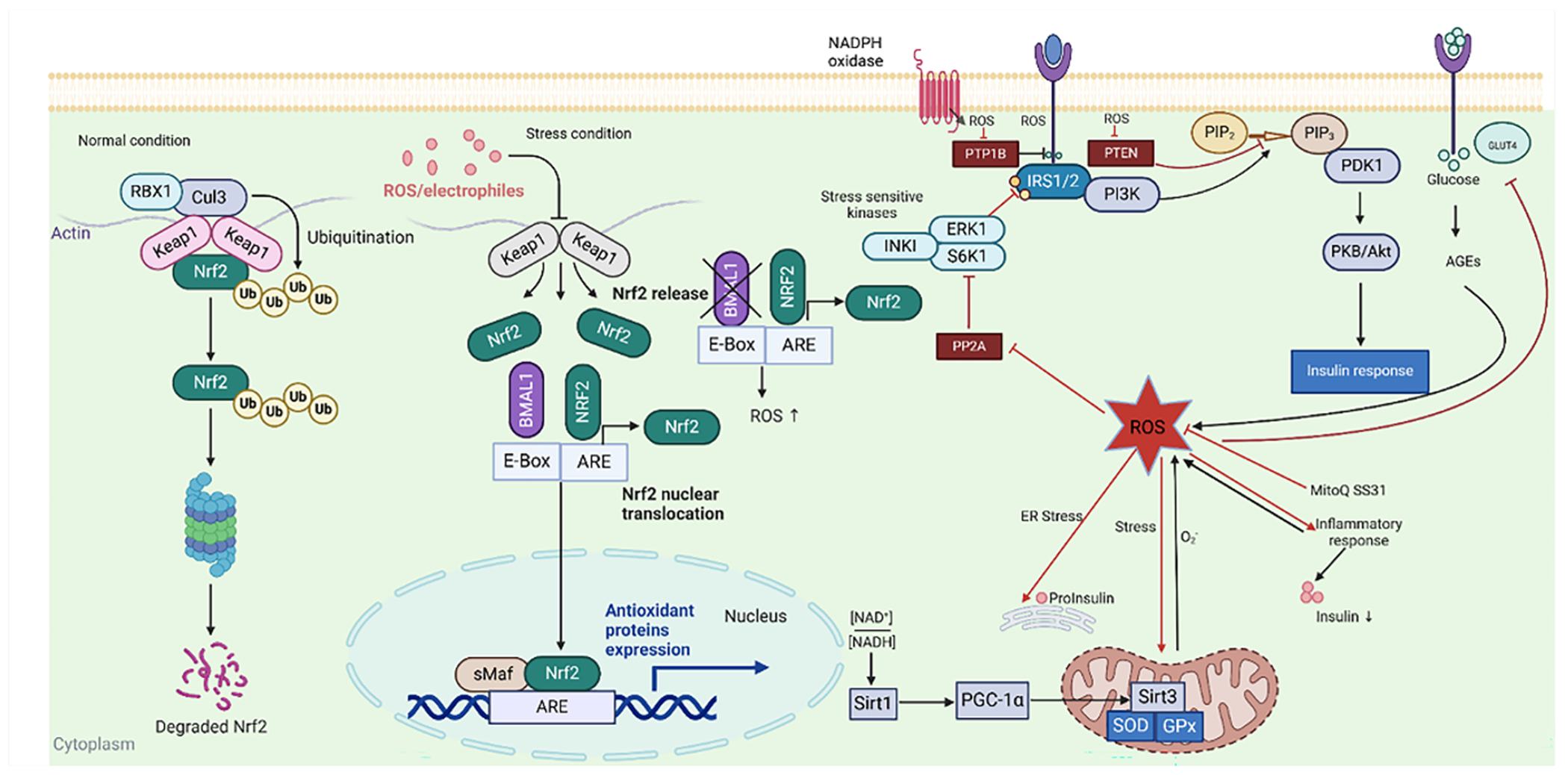
BMAL1-mediated regulation of keap1/Nrf2/ARE pathway in oxidative stress control and insulin signaling. Under normal conditions, cells continuously produce Nrf2, but it remains inactive in the cytoplasm due to its interaction with the inhibitory protein Keap1. Keap1 facilitates Nrf2 degradation by promoting its ubiquitination through its connection with the Cul3-Rbx1 E3 ubiquitin ligase complex. When cellular stress occurs, reactive cysteine residues in Keap1 undergo modifications, triggering structural changes that prevent Nrf2 degradation and allow its stabilization. In this process, BMAL1 binds to the antioxidant response element (ARE) via the E-box sequence on the Nrf2 promoter, leading to increased transcription and stabilization of Nrf2. Upon activation, Nrf2 is translocated to the nucleus, where it pairs with small Maf proteins to form a heterodimer and regulate the expression of antioxidant genes. The NRF2-sMaf complex binds to genes in the nucleus that contain antioxidant response elements (ARE) and are responsible for regulating metabolism, detoxification, antioxidant defense, and anti-inflammatory processes. Disruption of the circadian rhythm due to BMAL1 loss impairs the Nrf2 pathway, leading to cellular damage. Reactive oxygen species (ROS) relieve the inhibition of insulin receptors by phosphatases like PTP1B, allowing cells to respond to insulin signaling. For example, increased mitochondrial respiration elevates ROS production, shifting the cellular environment to a more oxidative state. This oxidative shift activates stress-sensitive kinases by inhibiting redox-sensitive phosphatases. These kinases, in turn, phosphorylate IRS proteins in an inhibitory manner, disrupting signal amplification. Mitochondrial antioxidants such as SS31 and MitoQ, along with the PGC-1α-dependent detoxification system involving Sirt3, SOD, and GPx, help regulate mitochondrial ROS levels. Elevated levels of ROS can contribute to insulin resistance by disrupting key insulin signaling pathways, including Akt, PI3K, and JNK. Additionally, ROS can impair the function of cellular organelles, leading to reduced energy production and the activation of apoptotic processes.

The pathophysiology and pathogenesis of several disease conditions, including diabetes, involve oxidative stress. At low concentrations, reactive oxygen species (ROS) function as signaling molecules that support normal insulin signaling. However, when ROS levels become excessive, they induce oxidative stress, which interferes with insulin receptor activity and downstream signaling pathways, potentially contributing to insulin resistance, thus demonstrating a nuanced, context-dependent interaction between ROS and insulin signaling (Figure 4). Oxidative stress is an endogenous biochemical process that is strongly influenced by the biological clocks and occurs due to an imbalance between ROS and endogenous antioxidant production. This is seen in disease conditions where the body’s ability to detoxify these reactive intermediates is impaired. Circadian regulation of oxidative stress and its impact on diabetes varies in various organs and systems, hence affecting disease progression. For instance, the insulin-producing pancreatic β-cells are susceptible to oxidative damage because of their low expression of protective antioxidants. The majority of protective antioxidant enzymes are regulated by *Nrf2*. This gene is shown to be rhythmically expressed under the influence of *Bmal1* in the pancreatic clock [123] as well as other tissues [124]. This may in part explain the circadian rhythm of oxidative stress in the pancreas, which typically peaks during the active phase. Exacerbation of oxidative stress through circadian disruption in the pancreatic β-cells predisposes to impaired insulin production and β-cells death as seen in diabetic patients. In the liver, the rhythmic expression of oxidative stress is closely linked to feeding cycles. During the activity period, ROS levels peak due to increased metabolic activities, especially during high nutrient consumption. The circadian clock regulates the peak and trough of endogenous antioxidant enzymes. For instance, antioxidants such as catalase (CAT) and superoxide dismutase (SOD) are expressed in a diurnal fashion in the mouse liver [125]. This indicates that there is variation in how the body responds to oxidative stress within a day. Moreso, with disruption of the circadian system or during a high-fat diet, the ability of the liver to mitigate oxidative stress is reduced. Consequently, lipid peroxidation occurs and is characterized by high malondialdehyde (MDA) levels. This is followed by inflammation and impaired insulin response, all of which are reminiscent of non-alcoholic fatty liver disease (NAFLD) and type 2 diabetes.

The skeletal muscle plays a central role in glucose metabolism and is particularly susceptible to oxidative stress during periods of physical activity. During high activity periods and energy expenditure, there are increased levels of circulating ROS. These activities are also controlled by the circadian clock within the skeletal muscles. To control oxidative stress, the circadian clock in muscle tissues synchronizes the expression of genes related to antioxidant defense and mitochondrial function [126]. Disruption of circadian rhythm, however, can also cause excessive oxidative stress in skeletal muscle, thereby altering insulin signaling and increasing susceptibility to insulin resistance. Another area where circadian modulation of oxidative stress is crucial is in adipose tissue, especially visceral fat. The balance between the processes that produce ROS, lipogenesis, and lipolysis is influenced by the circadian cycle in adipose tissue. Higher ROS production during the active phase is caused by increased lipolysis, which is usually annulled by antioxidant systems that are controlled by circadian rhythm [97]. Moreso, the circadian regulation of oxidative stress is frequently disturbed by obesity and metabolic syndrome, which results in chronic inflammation and insulin resistance in adipose tissue, both of which are important factors in the development of diabetes.

In general, the maintenance of metabolic balance in different organs and tissues depends critically on the circadian regulation of oxidative stress. These rhythms are important in several pathways in the pathogenesis and pathophysiology of diabetes. Circadian misalignment increases oxidative stress and, in turn, causes chronic inflammation, insulin resistance, and dysfunction of insulin-producing β-cells. Potential therapeutic targets for the prevention or management of diabetes are provided by an understanding of the circadian dynamics of oxidative stress in various organs, especially through lifestyle measures that preserve or restore circadian integrity.

## 5. Maternal Circadian Health and Generational Reprogramming

With the ongoing global pandemic of diabetes, there is an urgent need to expand the investigation of its etiological risk factors. The developmental origins of health and disease (DOHaD) concept posits that predisposition to disease in adulthood is in part determined by factors acting during prenatal development [127]. While much research into DOHaD looks at factors such as maternal smoking, psychosocial stressors, chemicals, and pollutants [128], or maternal obesity and metabolic diseases increase predisposition to offspring obesity and metabolic disease, one emerging avenue is maternal circadian health [112,129], which means keeping the body’s internal 24 h clock, or circadian rhythm, aligned with the external light-dark cycle for optimal function, influencing sleep, hormones, metabolism, and immunity. Modern lifestyles are characterized by overexposure to artificial light, jet lag, shift work, and insomnia. Accumulating evidence supports that when pregnant mothers are exposed to such environments, lifestyles, and circumstances, the detrimental health consequences can be transgenerational [112,127–129].

The health and habits of a mother before and during gestation can have significant implications on the health outcomes of the offspring, not only during the gestational period, but also throughout the offspring’s lifespan. One of the most comprehensive analyses of maternal health factors resulting in offspring health outcomes is from the Dutch Famine of 1944–1945, where mothers who experienced malnutrition throughout the famine gave birth to offspring who not only expressed disturbance in cardiovascular, metabolic, and mental health throughout their lifetime, but their offspring experienced many of the same health problems as well [130]. While the downstream effects of nutritional malnourishment in pregnant mothers have been thoroughly researched, circadian malnourishment is less documented. Circadian disruption in humans can be caused by a variety of events, namely shift work, poor sleep health, jet lag, and excessive light at night[131]. In pregnant mothers, the effects of circadian disruption may not stop at the mother herself but be passed on to her offspring as well. Virtually all physiological processes, including those involved in materno-fetal communications, are regulated by the circadian clock. Circadian disruption in the mother will lead to desynchronization and malfunction of many of these processes, leading to either malnutrition or overnutrition of the fetus and epigenetic reprogramming via mechanisms requiring further investigation.

### 5.1. Maternal Circadian Health and Gestation

The mother communicates with the fetus via hormones, and in the scope of the circadian rhythm, the two that set the stage are melatonin and glucocorticoids [132,133]. Melatonin is responsible for the transmission of the circadian rhythm from the mother to the offspring, establishing fetal circadian rhythmicity. Prior to fetal SCN development, maternal melatonin freely crosses into the placenta and acts on melatonin receptors in peripheral tissues and the offspring’s developing SCN to set the pace for the offspring [132–134]. When melatonin crosses into the placenta, it binds to melatonin receptors MT1 and MT2, G protein-coupled receptors (GPCRs) that inhibit adenylate cyclase to decrease cyclic adenosine monophosphate (cAMP) levels [135]. This drop in cAMP serves as a molecular signal that influences the phosphorylation of cAMP response element-binding protein (CREBP), which then binds to a cAMP response element (CRE) on Per1’s gene promoter, thus resetting the cellular clocks in the absence of a fully developed SCN [135–137]. While melatonin signals when it is time for fetal organs to assume roles played during sleep, in the absence of a developed SCN, maternal glucocorticoids serve as an activating signal for peripheral oscillators. Once glucocorticoids from the mother cross into fetal tissues, they bind to glucocorticoid response elements (GREs) that bind to Per1 and Per2 promoters for a rapid spike, as well as Bmal1 promoters that maintain a more enduring transcription of Bmal1 [138].

One key difference between melatonin and glucocorticoids is the role that 11-beta hydroxysteroid dehydrogenase type-2 (11β-HSD2) plays in transmission. Melatonin can pass freely from the mother to the fetus through the placenta [139]. Once in the placenta, melatonin can increase the activity of 11β-HSD2 to shield the placenta from either receiving too much maternal cortisol or cortisol at a circadian misaligned time [140]. This primarily occurs during the first two trimesters, as glucocorticoids play a greater role in the development of the liver in the third trimester [141]. In ex vivo human placentas, maternal glucocorticoids, however, are impeded from accessing the fetus by 11β-HSD2, up to as much as 80–90% [142], with the fetal HPA axis producing de novo cortisol after the 28th week of pregnancy [143].

### 5.2. Offspring Metabolic Reprogramming

**Figure 5.**
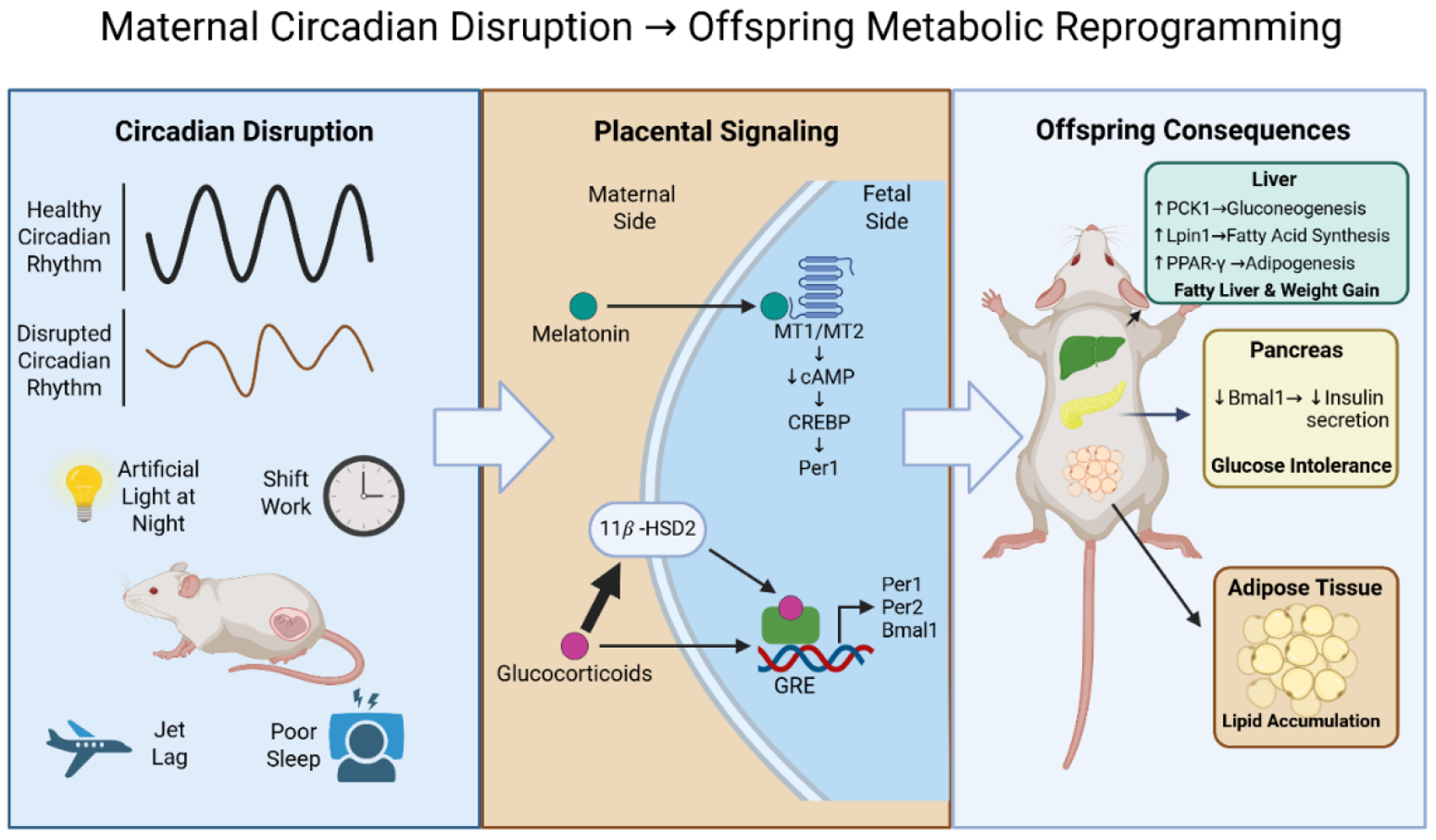
Maternal circadian disruption drives offspring metabolic reprogramming via placental signaling. (**Left**) Circadian disruption in pregnant mothers from artificial light at night, shift work, jet lag, or poor sleep alters endogenous rhythms of melatonin and glucocorticoids. (**Center**) Maternal melatonin freely crosses the placenta and binds fetal MT1/MT2 receptors, reducing cAMP and driving CREBP-mediated *Per1* transcription, while placental 11β-HSD2 normally inactivates most maternal glucocorticoids before they reach the fetus. Under circadian disruption, diminished melatonin reduces 11β-HSD2 activity, allowing excess glucocorticoids to enter fetal circulation and bind GREs that upregulate *Per1*, *Per2*, and *Bmal1*. (**Right**) This glucocorticoid oversaturation reprograms offspring metabolism: in the liver, increased *Pck1*, *Lpin1*, and PPAR-γ expression promotes gluconeogenesis, fatty acid synthesis, and adipogenesis. In the pancreas, impaired *Bmal1* reduces insulin secretion and glucose tolerance. In adipose tissue, lipid accumulation increases.

When a pregnant mother is subject to circadian disruption, the physiological effects can extend to her offspring. By means of a mistimed light environment, jet lag, or any of the other modalities of circadian disruption previously discussed, a mother’s natural pulses of melatonin and glucocorticoids can be disturbed [144]. By proxy, these extend to the fetus and manifest on a molecular, epigenetic, cellular, organ, and systemic level (Figure 5). On a molecular level, there would be less melatonin circulating to be picked up by the fetus. Because there is less binding of MT1/MT2, there would be less significant binding of cAMP to transcribe Per1 in the fetus [135–137]. Alone, this causes circadian disruption in a fetus that does not have its own SCN developed yet, but the other effect this has is that reduced melatonin would result in decreased 11β-HSD2 activation [145]. When maternal glucocorticoids are already elevated, and 11β-HSD2 is less active, there has been shown to be an oversaturation of glucocorticoids that enter fetal circulation [146,147]. With an overabundance of glucocorticoids, the first indications of metabolic dysfunction emerge in the fetus. It has been found that the GRE binds to a Pck1 promoter, transcribing phosphoenolpyruvate carboxylase 1 in the liver [148]. Pck1 promotes gluconeogenesis, thus predisposing the fetus to elevated glucose production [148]. The GREs will also bind to the Lpin1 promoter, encoding lipin-1, which promotes increased fatty acid synthesis, predisposing to further obesity, insulin resistance, and metabolic disease [149,150].

In the liver, these genetic alterations manifest as metabolic reprogramming. It was found that after the offspring were born, and into adulthood, they experienced challenges adapting to a high-fat diet and out-of-phase eating habits [129,151]. Additionally, genes that were found to play a part in fatty acid elongation, synthesis, and oxidation, such as Lipn1 and PPAR-γ, all became rhythmically dysregulated, further adding predisposition to lipid accumulation. Altogether, this resulted in an increase in body weight, an increase in liver weight, and an increase in the accumulation of lipids [129,150–152]. An interesting note regarding the liver is that not all DNA methylation enzymes were activated. Some key methylators, such as Mat1a and Dnmt, were decreased in the fetal liver. However, in one study looking at the effects of exogenous melatonin administration on pregnant rodents, 60% of the genes affected by circadian disruptions were restored to their original function [153]. This study sought to find the connection between maternal circadian disruption and the downstream epigenetic effects on the offspring in rodents, as well as the effects of administration of exogenous melatonin to the pregnant mother on the offspring [153]. The study revealed that female offspring born under conditions of circadian-disrupted mothers suffered from hormone imbalances, chronic inflammation, and heart and kidney issues stemming from genetic alterations made in utero. Upon exogenous melatonin administration to the mother, however, many of these issues in the offspring were prevented, and nearly 60% of the genes altered by circadian disruption were “rescued” in utero, leading to better health outcomes as an adult [153]. These findings highlight the consequence of decreased endogenous production of melatonin by mothers because of circadian disruption, as well as exogenous melatonin possibly serving as a therapeutic intervention for circadian-disrupted mothers. However, more research is required, as imbalances of other maternal hormones may play a role in the genes not rescued by melatonin.

In the pancreas, the insulin-producing *β*-cells appear to show long-term impairment due to maternal circadian disruption. In an offspring that does not have a properly functioning SCN through maternal entrainment, *Bmal1* transcription may be impaired as a result. In looking at Bmal1 knockout mice, glucose-stimulated insulin secretions are diminished, pointing to *β*-cell function as a downstream target of such maternal disruption [154]. The prenatal period in rodents was found to be remarkably important, as mothers who experienced circadian disruption for the first 18 days of pregnancy gave birth to offspring who experienced poor glucose tolerance for the rest of their lives [155].

### 5.3. Sexual and Dietary Differences in Offspring Responses

Differences have been found between male and female offspring, as well as between standard diets and high-fat diets. When comparing males and females of circadian-disrupted mothers on normal diets, both showed normal weight; however, the females developed insulin resistance and glucose intolerance, while the male mice did not. However, switching over to a high-fat diet, the male mice showed increased body weight gain while the females did not [112]. A separate study shared similar results on high-fat diets, with males developing fatty liver and adipocyte hypertrophy, while females did so as well, but to a lesser extent [129]. It is worth noting that the maternal diet may play a role as well. In rodent mothers who ate a high-fat diet, male offspring showed modulation in genes relating to hunger, namely decreased expression of appetite suppressant POMC, as well as increased expression of appetite stimulant ApRG [156]. This study examined male offspring, as estrogen in females is known to influence food intake [157,158].

Another possible explanation for the sexual dimorphism may involve sex-specific epigenetic mechanisms. Females tend to maintain higher levels of O-GlcNAc transferase (OGT), which maintains higher levels of a repressive epigenetic modification, H3K27me3, thus protecting them more from prenatal insults [159]. OGT is translated from the X-linked gene *Ogt, which* plays a role in the utilization of cellular nutrients such as glucose, glutamine, and ATP by modifying the proteins that utilize those nutrients [159]. Another action of OGT is the stabilization of H3K27me3, a repressive histone methyltransferase. This increased production of H3K27me3 serves as a buffer between maternal stress signals and the fetal epigenetic profile [159]. Females produce higher levels of OGT because they have two X chromosomes, and while X-inactivation usually silences the inactive X chromosome, certain genes, such as *Ogt*, escape this silencing [160]. While the presence of estrogen as well as elevated OGT in females offers possible explanations for the sexual dimorphism between males and females, further research is required to observe the multitude of confounding variables, such as a closer look at hormonal and epigenetic differences that affect the development of offspring.

## 6. Chronotherapy of Diabetes

The goal of managing diabetes mellitus is to maintain blood glucose levels as close to normal as possible. This approach aims to delay the onset and progression of diabetic complications and improve overall prognosis, thereby enhancing quality of life and general well-being. In recent decades, chronotherapy has gained attention as a strategy for diabetes management. This approach involves timing drug treatments to align with the body’s natural rhythms that play a role in the pathophysiology of diabetes. In this case, the efficacy of therapeutic agents is improved while minimizing known and potential side effects. The importance of chronotherapy was demonstrated in a time-dependent insulin administration, where administration at ZT14 and ZT16 restored the compromised peripheral clock functions and ameliorated diabetes symptoms in a streptozotocin-treated diabetic mouse model [161]. This may also suggest that the timing of insulin administration within the peak of insulin sensitivity will provide a better prognosis for diabetes. Similarly, it has been shown that evening administration of metformin within the peak of hepatic gluconeogenesis improves glycemic control in contrast to morning dosing. This treatment must also consider the circadian variation in the pharmacokinetics of metformin, which has been established [162].

The hypothalamic SCN circadian clock, together with peripheral clocks in key metabolic tissues, plays a crucial role in maintaining proper blood glucose regulation. Disrupting these clocks by inhibiting core clock genes has been implicated in the development, progression, and long-term consequences of diabetes. One potential strategy for managing diabetes is to synchronize the central and peripheral clocks by manipulating biological timing. Maintaining glycemic status requires both exogenous medication and improved melatonin rhythm. Patients with diabetes may benefit from melatonin therapy in addition to other benefits since it may enhance β-cell activity, adiposity, synchronize the circadian rhythm with external cues, improve insulin response, and decrease the rate at which the disease progresses [163]. Medications that target *ROR*, *Crys*, and *Rev-erbs* are viable options for the treatment of metabolic disorders [164–166].

To prevent gluconeogenesis, synthetic *Cry1/2* agonists are employed [161,164]. Studies have shown that deploying drugs or phytochemicals such as biochennin and nobiletin in a chronopharmacological manner may offer chronotherapeutic benefits in the management of diabetes (Table 2). These agents are suggested to display circadian-dependent therapeutic outcomes by modulating the circadian oscillation of the core clock gene *Bmal1,* enhancing its amplitude and maintaining energy balance, as well as synchronizing peripheral clocks/target tissues by sending circadian signals [167,168]. Although the difficulties of contemporary life may persist, they can be effectively controlled to better synchronize with our circadian rhythms. Lifestyle interventions and circadian alignment can be a strategy to promote health. For example, consumption of carbohydrate-rich meals during the day may have less deleterious effects on glycemic levels when compared to nighttime consumption. Light therapy and melatonin administration may also provide the desired therapeutic outcome for shift workers and individuals who do not achieve the recommended 7–8 h of sleep per night. It is also important to align their circadian rhythm with their diet and lifestyle choices. Moreso, research is ongoing to determine optimal feeding schedules, even among individuals who work regular day shifts. Hence, aside from conventional chronotherapy with antidiabetic agents, circadian rhythm-mediated lifestyle modification is an important therapeutic option for diabetes management. Glycemic control can be improved by maintaining a healthy sleeping pattern, an adequate feeding schedule (chrono-nutrition), and the proper timing of exercise (chrono-exercise). Furthermore, maintaining a healthy circadian rhythm can be aided by limiting light exposure at night and maximizing natural light exposure during the day, which may lower the risk of diabetes.

## 7. Conclusions

The circadian system regulates 24 h physiological cycles that are necessary to preserve general health. Among the vital functions controlled by this system is the homeostasis of glucose. Disturbances from typical circadian rhythms, which can cause these processes to become misaligned, are increasingly associated with negative health outcomes, such as the initiation and development of type 2 diabetes mellitus. In this review, we examined the relationship between the circadian system and glucose metabolism. We defined the hierarchy of the circadian system and showed evidence of circadian involvement in glucose homeostasis. By showing the functions of peripheral clocks and the core clock genes in glucose control, it can be further appreciated that drugs targeting these molecular pathways may provide benefits in the management of type 2 diabetes. Moreso, further research is needed to elucidate and maximize to its full potential the exact molecular mechanisms of the circadian clock dysfunction and its involvement in diabetes. This will further identify potential points for therapeutic intervention for the management of type 2 diabetes mellitus.

## Figures and Tables

**Table 1.** Experimental studies on the impact of clock gene mutations on glucose homeostasis in mice.

Model	Clock Genes	Body Weight	White Adipose Tissue	Blood Glucose	Glucose Tolerance	Insulin	Insulin Sensitivity	Lipids	Ref.
*Bmal1* ^−/−^	*Bmal1*	Increased		Varies based on time of day		Normal	Increased	Increased	[36]
*PdxCre Bmal1^flx/flx^*	*Bmal1*	Normal		Elevated	Impaired	Decreased			[37]
*Mlc1f–Cre Bmal1 ^flx/flx^*	*Bmal1*	Increased			Normal global glucose tolerance		Increased muscle insulin resistance		[38]
*iMSBmal1^−/−^*	*Bmal1*	Decreased	Decreased	Increased	Decreased	Increased			[39]
*Bmal1* null	*Bmal1*	Reduced	Increased	Normal	Normal	Decreased	Normal	Normal	[40]
*Clock^mut^*	*Clock*	Increased	Normal	Decreased			Increased		[36]
*Clock* ^Δ*19*/Δ*19*^	*Clock*			Elevated	Impaired	Decreased	Normal		[37]
*Clock^−/−^*	*Clock*	Increased		Elevated		Normal		Increase	[41]
Clock^−/−^	*Clock*	Reduced		Normal		Normal		Decrease	[42]
Clock^−/−^	*Clock*			Normal		Normal			[43]
*Cry1^−/−^; Cry2^−/−^*	*Cry1/Cry2*			Increased	Decreased	Normal	Normal		[44]
*Cry1/2* ^−/−^	*Cry1/Cry2*	Decreased		Increased		Increased	Decreased		[45]
*Per2^Brdm1^*	*Per2*			Decreased					[46]
*Per2^−/−^*	*Per2*	Decreased	Decreased					Decreased	[47]
*mPer2*-null	*Per2*			Normal	Increased	Increased	Increased		[48]
*Per2^Brdm1^*	*Per2*	Normal		Decreased	Normal		Normal	Decreased	[49]
*Per2^Brdm1^*	*Per2*	Normal						Normal	[12]
*Per2^Brd^*	*Per2*	Normal		Normal	Increased				[50]
*Per1^Brd^*	*Per1*	Decreased		Decreased	Increased				[50]
*Rev-erb-α*^−/−^ *Rev-erb-β*^−/−^	*Rev-erbα/β*			Increased				Decreased	[51]
*Rev-erb-α* ^−/−^	*Rever-bα/β*	Normal	Increased	Increased	Normal		Normal	Decreased	[52]
*Rev-erb-α* ^−/−^	*Rever-bα/β*	Normal		Normal	Decreased	Normal	Decreased		[53]

**Table 2.** Experimental studies on the regulation of glucose metabolism by targeting the circadian clock.

Phytochemical Agent	Model	Circadian Target/Tissues	Metabolic Processes	Mechanism	Ref.
TW68	HepG2 C57BL/6J mice	*Cry1* and *Cry2*	Stabilizes *Crys* and reduces gluconeogenesis	Decrease *PCK1* and *G6PC*	[169]
Nobiletin	*db*/*db* obese mice	Hepatocytes *Clock*	Energy regulation	enhancing amplitude and energy balance	[167]
Nobiletin	C57BL/6J mice	Adipocytes *Bmal1*	Lipolysis	Increases ATGL levels via RORα/γ activity	[168]
Nobiletin	HepG2 cells	Liver	Increased insulin sensitivity/Lipid metabolism	Circadian clock reprogramming	[170]
(−)-Epigallocatechin-3-gallate	HepG2 cells	Hepatic *Bmal1*	Gluconeogenesis	Enhances insulin signaling pathways for AMPK and IRS/AKT/GLUT2	[171]
KL001	C57BL/6J	Liver/*Cry1/2*	Gluconeogenesis	Inhibition of basal production	[164]
Hesperidin	*Drosophila melanogaster*	Global Cry^b^ mutants	Oxidative stress	Restores antioxidant rhythms	[172]
Genistein	C57BL/6J mice	Hypothalamus	Glucose metabolism	Circadian entrainment via *Per1*, *c-Fos*, and *Grin1*	[173]
Resveratrol	C57BL/6 mice	Liver	Hepatic glucose metabolism	Circadian entrainment of BMAL1 via SIRT1	[174]

## Data Availability

No new data were created or analyzed in this study. Data sharing is not applicable to this article.

## References

[1] American Diabetes Association Professional Practice Committee. 2. Diagnosis and Classification of Diabetes: Standards of Care in Diabetes—2024. Diabetes Care 2023, 47, S20–S42. 10.2337/dc24-S002. PMC10725812 38078589

[2] Cho NH; Shaw JE; Karuranga S; Huang Y; da Rocha Fernandes JD; Ohlrogge AW; Malanda B. IDF Diabetes Atlas: Global estimates of diabetes prevalence for 2017 and projections for 2045. Diabetes Res. Clin. Pract. 2018, 138, 271–281. 10.1016/j.diabres.2018.02.023. 29496507

[3] Centers for Disease Control and Prevention. National Diabetes Statistics Report; CDC: Atlanta, GA, USA, 2026. Available online: https://www.cdc.gov/diabetes/communication-resources/diabetes-statistics.html (accessed on 22 June 2026).

[4] Sheehan CM; Frochen SE; Walsemann KM; Ailshire JA. Are U.S. adults reporting less sleep?: Findings from sleep duration trends in the National Health Interview Survey, 2004–2017. Sleep 2019, 42, zsy221. 10.1093/sleep/zsy221. 30452725 PMC6941709

[5] Roenneberg T; Allebrandt KV; Merrow M; Vetter C. Social jetlag and obesity. Curr. Biol. 2012, 22, 939–943. 10.1016/j.cub.2012.03.038. 22578422

[6] Vetter C; Dashti HS; Lane JM; Anderson SG; Schernhammer ES; Rutter MK; Saxena R; Scheer F. Night Shift Work, Genetic Risk, and Type 2 Diabetes in the UK Biobank. Diabetes Care 2018, 41, 762–769. 10.2337/dc17-1933. 29440150 PMC5860836

[7] Xie F; Hu K; Fu R; Zhang Y; Xiao K; Tu J. Association between night shift work and the risk of type 2 diabetes mellitus: A cohort-based meta-analysis. BMC Endocr. Disord. 2024, 24, 268. 10.1186/s12902-024-01808-w. 39696306 PMC11653577

[8] Akerstedt T; Gillberg M. The circadian variation of experimentally displaced sleep. Sleep 1981, 4, 159–169. 10.1093/sleep/4.2.159. 7256076

[9] Schulz P. Biological clocks and the practice of psychiatry. Dialogues Clin. Neurosci. 2007, 9, 237–255. 10.31887/DCNS.2007.9.3/pschulz. 17969862 PMC3202493

[10] Redfern PH; Waterhouse JM; Minors DS. Circadian rhythms: Principles and measurement. Pharmacol. Ther. 1991, 49, 311–327. 10.1016/0163-7258(91)90061-p. 2052628

[11] Kalsbeek A; Strubbe JH. Circadian control of insulin secretion is independent of the temporal distribution of feeding. Physiol. Behav. 1998, 63, 553–558. 10.1016/s0031-9384(97)00493-9. 9523898

[12] Chappuis S; Ripperger JA; Schnell A; Rando G; Jud C; Wahli W; Albrecht U. Role of the circadian clock gene Per2 in adaptation to cold temperature. Mol. Metab. 2013, 2, 184–193. 10.1016/j.molmet.2013.05.002. 24049733 PMC3773826

[13] Johnston JD; Ordovás JM; Scheer FA; Turek FW. Circadian Rhythms, Metabolism, and Chrononutrition in Rodents and Humans. Adv. Nutr. 2016, 7, 399–406. 10.3945/an.115.010777. 26980824 PMC4785478

[14] Reppert SM; Weaver DR. Coordination of circadian timing in mammals. Nature 2002, 418, 935–941. 10.1038/nature00965. 12198538

[15] Kalsbeek A; Palm IF; La Fleur SE; Scheer FA; Perreau-Lenz S; Ruiter M; Kreier F; Cailotto C; Buijs RM. SCN outputs and the hypothalamic balance of life. J. Biol. Rhythm. 2006, 21, 458–469. 10.1177/0748730406293854. 17107936

[16] Okamura H. Suprachiasmatic nucleus clock time in the mammalian circadian system. Cold Spring Harb. Symp. Quant. Biol. 2007, 72, 551–556. 10.1101/sqb.2007.72.033. 18419314

[17] Hattar S; Lucas RJ; Mrosovsky N; Thompson S; Douglas RH; Hankins MW; Lem J; Biel M; Hofmann F; Foster RG; Melanopsin and rod-cone photoreceptive systems account for all major accessory visual functions in mice. Nature 2003, 424, 76–81. 10.1038/nature01761. 12808468 PMC2885907

[18] Chen R; Seo DO; Bell E; von Gall C; Lee C. Strong resetting of the mammalian clock by constant light followed by constant darkness. J. Neurosci. 2008, 28, 11839–11847. 10.1523/jneurosci.2191-08.2008. 19005049 PMC2626189

[19] Berson DM; Dunn FA; Takao M. Phototransduction by retinal ganglion cells that set the circadian clock. Science 2002, 295, 1070–1073. 10.1126/science.1067262. 11834835

[20] Freedman MS; Lucas RJ; Soni B; von Schantz M; Muñoz M; David-Gray Z; Foster R. Regulation of mammalian circadian behavior by non-rod, non-cone, ocular photoreceptors. Science 1999, 284, 502–504. 10.1126/science.284.5413.502. 10205061

[21] Peschke E; Peschke D. Evidence for a circadian rhythm of insulin release from perifused rat pancreatic islets. Diabetologia 1998, 41, 1085–1092. 10.1007/s001250051034. 9754828

[22] Takahashi JS. Transcriptional architecture of the mammalian circadian clock. Nat. Rev. Genet. 2017, 18, 164–179. 10.1038/nrg.2016.150. 27990019 PMC5501165

[23] Mason IC; Qian J; Adler GK; Scheer F. Impact of circadian disruption on glucose metabolism: Implications for type 2 diabetes. Diabetologia 2020, 63, 462–472. 10.1007/s00125-019-05059-6. 31915891 PMC7002226

[24] Van Cauter E; Polonsky KS; Scheen AJ. Roles of circadian rhythmicity and sleep in human glucose regulation. Endocr. Rev. 1997, 18, 716–738. 10.1210/edrv.18.5.0317. 9331550

[25] la Fleur SE; Kalsbeek A; Wortel J; Fekkes ML; Buijs RM. A daily rhythm in glucose tolerance: A role for the suprachiasmatic nucleus. Diabetes 2001, 50, 1237–1243. 10.2337/diabetes.50.6.1237. 11375322

[26] Bolli GB; Gottesman IS; Cryer PE; Gerich JE. Glucose counterregulation during prolonged hypoglycemia in normal humans. Am. J. Physiol. 1984, 247, E206–E214. 10.1152/ajpendo.1984.247.2.E206. 6380307

[27] Linkowski P; Kerkhofs M; Van Onderbergen A; Hubain P; Copinschi G; L’Hermite-Balériaux M; Leclercq R; Brasseur M; Mendlewicz J; Van Cauter E. The 24-hour profiles of cortisol, prolactin, and growth hormone secretion in mania. Arch. Gen. Psychiatry 1994, 51, 616–624. 10.1001/archpsyc.1994.03950080028004. 8042910

[28] Linkowski P; Mendlewicz J; Kerkhofs M; Leclercq R; Golstein J; Brasseur M; Copinschi G; Van Cauter E. 24-hour profiles of adrenocorticotropin, cortisol, and growth hormone in major depressive illness: Effect of antidepressant treatment. J. Clin. Endocrinol. Metab. 1987, 65, 141–152. 10.1210/jcem-65-1-141. 3034952

[29] Peng F; Li X; Xiao F; Zhao R; Sun Z. Circadian clock, diurnal glucose metabolic rhythm, and dawn phenomenon. Trends Neurosci. 2022, 45, 471–482. 10.1016/j.tins.2022.03.010. 35466006 PMC9117496

[30] Ahmed M; Gannon MC; Nuttall FQ. Postprandial plasma glucose, insulin, glucagon and triglyceride responses to a standard diet in normal subjects. Diabetologia 1976, 12, 61–67. 10.1007/bf01221966. 943353

[31] Carroll KF; Nestel PJ. Diurnal variation in glucose tolerance and in insulin secretion in man. Diabetes 1973, 22, 333–348. 10.2337/diab.22.5.333. 4700047

[32] Jarrett RJ; Baker IA; Keen H; Oakley NW. Diurnal variation in oral glucose tolerance: Blood sugar and plasma insulin levels morning, afternoon, and evening. Br. Med. J. 1972, 1, 199–201. 10.1136/bmj.1.5794.199. 5058728 PMC1789199

[33] Strubbe JH; Steffens AB; De Ruiter L. Plasma insulin and the time pattern of feeding in the rat. Physiol. Behav. 1977, 18, 81–86. 10.1016/0031-9384(77)90097-X. 561972

[34] Hara E; Saito M. Diurnal changes in plasma glucose and insulin responses to oral glucose load in rats. Am. J. Physiol. 1980, 238, E463–E466. 10.1152/ajpendo.1980.238.5.E463. 6990785

[35] Arble DM; Holland J; Ottaway N; Sorrell J; Pressler JW; Morano R; Woods SC; Seeley RJ; Herman JP; Sandoval DA; The melanocortin-4 receptor integrates circadian light cues and metabolism. Endocrinology 2015, 156, 1685–1691. 10.1210/en.2014-1937. 25730108 PMC4398770

[36] Rudic RD; McNamara P; Curtis AM; Boston RC; Panda S; Hogenesch JB; Fitzgerald GA. BMAL1 and CLOCK, two essential components of the circadian clock, are involved in glucose homeostasis. PLoS Biol. 2004, 2, e377. 10.1371/journal.pbio.0020377. 15523558 PMC524471

[37] Marcheva B; Ramsey KM; Buhr ED; Kobayashi Y; Su H; Ko CH; Ivanova G; Omura C; Mo S; Vitaterna MH; Disruption of the clock components CLOCK and BMAL1 leads to hypoinsulinaemia and diabetes. Nature 2010, 466, 627–631. 10.1038/nature09253. 20562852 PMC2920067

[38] Dyar KA; Ciciliot S; Wright LE; Biensø RS; Tagliazucchi GM; Patel VR; Forcato M; Paz MI; Gudiksen A; Solagna F; Muscle insulin sensitivity and glucose metabolism are controlled by the intrinsic muscle clock. Mol. Metab. 2014, 3, 29–41. 10.1016/j.molmet.2013.10.005. 24567902 PMC3929910

[39] Harfmann BD; Schroder EA; Kachman MT; Hodge BA; Zhang X; Esser KA. Muscle-specific loss of Bmal1 leads to disrupted tissue glucose metabolism and systemic glucose homeostasis. Skelet. Muscle 2016, 6, 12. 10.1186/s13395-016-0082-x. 27486508 PMC4969979

[40] Kennaway DJ; Varcoe TJ; Voultsios A; Boden MJ. Global loss of bmal1 expression alters adipose tissue hormones, gene expression and glucose metabolism. PLoS ONE 2013, 8, e65255. 10.1371/journal.pone.0065255. 23750248 PMC3672145

[41] Turek FW; Joshu C; Kohsaka A; Lin E; Ivanova G; McDearmon E; Laposky A; Losee-Olson S; Easton A; Jensen DR; Obesity and Metabolic Syndrome in Circadian Clock Mutant Mice. Science 2005, 308, 1043–1045. 10.1126/science.1108750. 15845877 PMC3764501

[42] Oishi K; Atsumi G; Sugiyama S; Kodomari I; Kasamatsu M; Machida K; Ishida N. Disrupted fat absorption attenuates obesity induced by a high-fat diet in Clock mutant mice. FEBS Lett. 2006, 580, 127–130. 10.1016/j.febslet.2005.11.063. 16343493

[43] Doi R; Oishi K; Ishida N. CLOCK regulates circadian rhythms of hepatic glycogen synthesis through transcriptional activation of Gys2. J. Biol. Chem. 2010, 285, 22114–22121. 10.1074/jbc.M110.110361. 20430893 PMC2903367

[44] Lamia KA; Papp SJ; Yu RT; Barish GD; Uhlenhaut NH; Jonker JW; Downes M; Evans RM. Cryptochromes mediate rhythmic repression of the glucocorticoid receptor. Nature 2011, 480, 552–556. 10.1038/nature10700. 22170608 PMC3245818

[45] Barclay JL; Shostak A; Leliavski A; Tsang AH; Jöhren O; Müller-Fielitz H; Landgraf D; Naujokat N; van der Horst GT; Oster H. High-fat diet-induced hyperinsulinemia and tissue-specific insulin resistance in Cry-deficient mice. Am. J. Physiol. Endocrinol. Metab. 2013, 304, E1053–E1063. 10.1152/ajpendo.00512.2012. 23531614

[46] Schmutz I; Ripperger JA; Baeriswyl-Aebischer S; Albrecht U. The mammalian clock component PERIOD2 coordinates circadian output by interaction with nuclear receptors. Genes Dev. 2010, 24, 345–357. 10.1101/gad.564110. 20159955 PMC2816734

[47] Grimaldi B; Bellet MM; Katada S; Astarita G; Hirayama J; Amin RH; Granneman JG; Piomelli D; Leff T; Sassone-Corsi P. PER2 Controls Lipid Metabolism by Direct Regulation of PPARγ. Cell Metab. 2010, 12, 509–520. 10.1016/j.cmet.2010.10.005. 21035761 PMC4103168

[48] Zhao Y; Zhang Y; Zhou M; Wang S; Hua Z; Zhang J. Loss of mPer2 increases plasma insulin levels by enhanced glucose-stimulated insulin secretion and impaired insulin clearance in mice. FEBS Lett. 2012, 586, 1306–1311. 10.1016/j.febslet.2012.03.034. 22504074

[49] Zani F; Breasson L; Becattini B; Vukolic A; Montani JP; Albrecht U; Provenzani A; Ripperger JA; Solinas G. PER2 promotes glucose storage to liver glycogen during feeding and acute fasting by inducing Gys2 PTG and G L expression. Mol. Metab. 2013, 2, 292–305. 10.1016/j.molmet.2013.06.006. 24049741 PMC3773838

[50] Dallmann R; Touma C; Palme R; Albrecht U; Steinlechner S. Impaired daily glucocorticoid rhythm in Per1Brdmice. J. Comp. Physiol. A 2006, 192, 769–775. 10.1007/s00359-006-0114-9. 16505983

[51] Cho H; Zhao X; Hatori M; Yu RT; Barish GD; Lam MT; Chong L-W; DiTacchio L; Atkins AR; Glass CK; Regulation of circadian behaviour and metabolism by REV-ERB-α and REV-ERB-β. Nature 2012, 485, 123–127. 10.1038/nature11048. 22460952 PMC3367514

[52] Delezie J; Dumont S; Dardente H; Oudart H; Gréchez-Cassiau A; Klosen P; Teboul M; Delaunay F; Pévet P; Challet E. The nuclear receptor REV-ERBα is required for the daily balance of carbohydrate and lipid metabolism. FASEB J. 2012, 26, 3321–3335. 10.1096/fj.12-208751. 22562834

[53] Ding G; Li X; Hou X; Zhou W; Gong Y; Liu F; He Y; Song J; Wang J; Basil P; REV-ERB in GABAergic neurons controls diurnal hepatic insulin sensitivity. Nature 2021, 592, 763–767. 10.1038/s41586-021-03358-w. 33762728 PMC8085086

[54] Lee A; Ader M; Bray GA; Bergman RN. Diurnal variation in glucose tolerance. Cyclic suppression of insulin action and insulin secretion in normal-weight, but not obese, subjects. Diabetes 1992, 41, 750–759. 10.2337/diab.41.6.750. 1587401

[55] Boden G; Ruiz J; Urbain JL; Chen X. Evidence for a circadian rhythm of insulin secretion. Am. J. Physiol. 1996, 271, E246–E252. 10.1152/ajpendo.1996.271.2.E246. 8770017

[56] Perelis M; Marcheva B; Ramsey KM; Schipma MJ; Hutchison AL; Taguchi A; Peek CB; Hong H; Huang W; Omura C; Pancreatic β cell enhancers regulate rhythmic transcription of genes controlling insulin secretion. Science 2015, 350, aac4250. 10.1126/science.aac4250. 26542580 PMC4669216

[57] Ruiter M; La Fleur SE; van Heijningen C; van der Vliet J; Kalsbeek A; Buijs RM. The daily rhythm in plasma glucagon concentrations in the rat is modulated by the biological clock and by feeding behavior. Diabetes 2003, 52, 1709–1715. 10.2337/diabetes.52.7.1709. 12829637

[58] Saad A; Dalla Man C; Nandy DK; Levine JA; Bharucha AE; Rizza RA; Basu R; Carter RE; Cobelli C; Kudva YC; Diurnal pattern to insulin secretion and insulin action in healthy individuals. Diabetes 2012, 61, 2691–2700. 10.2337/db11-1478. 22751690 PMC3478548

[59] Pulimeno P; Mannic T; Sage D; Giovannoni L; Salmon P; Lemeille S; Giry-Laterriere M; Unser M; Bosco D; Bauer C; Autonomous and self-sustained circadian oscillators displayed in human islet cells. Diabetologia 2013, 56, 497–507. 10.1007/s00125-012-2779-7. 23242133 PMC3563957

[60] Qian J; Block GD; Colwell CS; Matveyenko AV. Consequences of exposure to light at night on the pancreatic islet circadian clock and function in rats. Diabetes 2013, 62, 3469–3478. 10.2337/db12-1543. 23775768 PMC3781472

[61] Qian J; Yeh B; Rakshit K; Colwell CS; Matveyenko AV. Circadian Disruption and Diet-Induced Obesity Synergize to Promote Development of β-Cell Failure and Diabetes in Male Rats. Endocrinology 2015, 156, 4426–4436. 10.1210/en.2015-1516. 26348474 PMC4655211

[62] Petrenko V; Gosmain Y; Dibner C. High-Resolution Recording of the Circadian Oscillator in Primary Mouse α- and β-Cell Culture. Front. Endocrinol. 2017, 8, 68. 10.3389/fendo.2017.00068. PMC5383706 28439257

[63] Petrenko V; Saini C; Giovannoni L; Gobet C; Sage D; Unser M; Heddad Masson M; Gu G; Bosco D; Gachon F; Pancreatic α- and β-cellular clocks have distinct molecular properties and impact on islet hormone secretion and gene expression. Genes Dev. 2017, 31, 383–398. 10.1101/gad.290379.116. 28275001 PMC5358758

[64] Allaman-Pillet N; Roduit R; Oberson A; Abdelli S; Ruiz J; Beckmann JS; Schorderet DF; Bonny C. Circadian regulation of islet genes involved in insulin production and secretion. Mol. Cell. Endocrinol. 2004, 226, 59–66. 10.1016/j.mce.2004.06.001. 15489006

[65] Nakabayashi H; Ohta Y; Yamamoto M; Susuki Y; Taguchi A; Tanabe K; Kondo M; Hatanaka M; Nagao Y; Tanizawa Y. Clock-controlled output gene Dbp is a regulator of Arnt/Hif-1β gene expression in pancreatic islet β-cells. Biochem. Biophys. Res. Commun. 2013, 434, 370–375. 10.1016/j.bbrc.2013.03.084. 23567972

[66] Perrin L; Loizides-Mangold U; Skarupelova S; Pulimeno P; Chanon S; Robert M; Bouzakri K; Modoux C; Roux-Lombard P; Vidal H; Human skeletal myotubes display a cell-autonomous circadian clock implicated in basal myokine secretion. Mol. Metab. 2015, 4, 834–845. 10.1016/j.molmet.2015.07.009. 26629407 PMC4632112

[67] Hansen J; Timmers S; Moonen-Kornips E; Duez H; Staels B; Hesselink MK; Schrauwen P. Synchronized human skeletal myotubes of lean, obese and type 2 diabetic patients maintain circadian oscillation of clock genes. Sci. Rep. 2016, 6, 35047. 10.1038/srep35047. 27756900 PMC5069469

[68] Wefers J; Connell NJ; Fealy CE; Andriessen C; de Wit V; van Moorsel D; Moonen-Kornips E; Jörgensen JA; Hesselink MKC; Havekes B; Day-night rhythm of skeletal muscle metabolism is disturbed in older, metabolically compromised individuals. Mol. Metab. 2020, 41, 101050. 10.1016/j.molmet.2020.101050. 32659272 PMC7415921

[69] McGinnis GR; Young ME. Circadian regulation of metabolic homeostasis: Causes and consequences. Nat. Sci. Sleep 2016, 8, 163–180. 10.2147/nss.S78946. 27313482 PMC4890688

[70] Macauley M; Smith FE; Thelwall PE; Hollingsworth KG; Taylor R. Diurnal variation in skeletal muscle and liver glycogen in humans with normal health and Type 2 diabetes. Clin. Sci. 2015, 128, 707–713. 10.1042/cs20140681. 25583442

[71] van Moorsel D; Hansen J; Havekes B; Scheer F; Jörgensen JA; Hoeks J; Schrauwen-Hinderling VB; Duez H; Lefebvre P; Schaper NC; Demonstration of a day-night rhythm in human skeletal muscle oxidative capacity. Mol. Metab. 2016, 5, 635–645. 10.1016/j.molmet.2016.06.012. 27656401 PMC5021670

[72] Chang SW; Yoshihara T; Machida S; Naito H. Circadian rhythm of intracellular protein synthesis signaling in rat cardiac and skeletal muscles. Biochem. Biophys. Rep. 2017, 9, 153–158. 10.1016/j.bbrep.2016.12.005. 28956001 PMC5614553

[73] Perrin L; Loizides-Mangold U; Chanon S; Gobet C; Hulo N; Isenegger L; Weger BD; Migliavacca E; Charpagne A; Betts JA; Transcriptomic analyses reveal rhythmic and CLOCK-driven pathways in human skeletal muscle. eLife 2018, 7, e34114. 10.7554/eLife.34114. 29658882 PMC5902165

[74] Bunger MK; Wilsbacher LD; Moran SM; Clendenin C; Radcliffe LA; Hogenesch JB; Simon MC; Takahashi JS; Bradfield CA. Mop3 Is an Essential Component of the Master Circadian Pacemaker in Mammals. Cell 2000, 103, 1009–1017. 10.1016/S0092-8674(00)00205-1. 11163178 PMC3779439

[75] Kondratov RV; Kondratova AA; Gorbacheva VY; Vykhovanets OV; Antoch MP. Early aging and age-related pathologies in mice deficient in BMAL1, the core componentof the circadian clock. Genes Dev. 2006, 20, 1868–1873. 10.1101/gad.1432206. 16847346 PMC1522083

[76] McDearmon EL; Patel KN; Ko CH; Walisser JA; Schook AC; Chong JL; Wilsbacher LD; Song EJ; Hong HK; Bradfield CA; Dissecting the functions of the mammalian clock protein BMAL1 by tissue-specific rescue in mice. Science 2006, 314, 1304–1308. 10.1126/science.1132430. 17124323 PMC3756687

[77] Hodge BA; Wen Y; Riley LA; Zhang X; England JH; Harfmann BD; Schroder EA; Esser KA. The endogenous molecular clock orchestrates the temporal separation of substrate metabolism in skeletal muscle. Skelet. Muscle 2015, 5, 17. 10.1186/s13395-015-0039-5. 26000164 PMC4440511

[78] Pendergrass M; Bertoldo A; Bonadonna R; Nucci G; Mandarino L; Cobelli C; Defronzo RA. Muscle glucose transport and phosphorylation in type 2 diabetic, obese nondiabetic, and genetically predisposed individuals. Am. J. Physiol. Endocrinol. Metab. 2007, 292, E92–E100. 10.1152/ajpendo.00617.2005. 16896161

[79] Ando H; Ushijima K; Shimba S; Fujimura A. Daily Fasting Blood Glucose Rhythm in Male Mice: A Role of the Circadian Clock in the Liver. Endocrinology 2016, 157, 463–469. 10.1210/en.2015-1376. 26653333

[80] Phillips LJ; Berry LJ. Circadian rhythm of mouse liver phosphoenolpyruvate carboxykinase. Am. J. Physiol. 1970, 218, 1440–1444. 10.1152/ajplegacy.1970.218.5.1440. 4314570

[81] Leighton B; Kowalchuk JM; Challiss RA; Newsholme EA. Circadian rhythm in sensitivity of glucose metabolism to insulin in rat soleus muscle. Am. J. Physiol. 1988, 255, E41–E45. 10.1152/ajpendo.1988.255.1.E41. 3291620

[82] Silverstein RL; Febbraio M. CD36, a scavenger receptor involved in immunity, metabolism, angiogenesis, and behavior. Sci. Signal. 2009, 2, re3. 10.1126/scisignal.272re3. 19471024 PMC2811062

[83] Miyaoka K; Kuwasako T; Hirano K; Nozaki S; Yamashita S; Matsuzawa Y. CD36 deficiency associated with insulin resistance. Lancet 2001, 357, 686–687. 10.1016/s0140-6736(00)04138-6. 11247555

[84] Glazier AM; Scott J; Aitman TJ. Molecular basis of the Cd36 chromosomal deletion underlying SHR defects in insulin action and fatty acid metabolism. Mamm. Genome 2002, 13, 108–113. 10.1007/s00335-001-2132-9. 11889559

[85] Corpeleijn E; van der Kallen CJ; Kruijshoop M; Magagnin MG; de Bruin TW; Feskens EJ; Saris WH; Blaak EE. Direct association of a promoter polymorphism in the CD36/FAT fatty acid transporter gene with Type 2 diabetes mellitus and insulin resistance. Diabet. Med. 2006, 23, 907–911. 10.1111/j.1464-5491.2006.01888.x. 16911630

[86] Chen M; Zhang Y; Zeng S; Li D; You M; Zhang M; Wang Z; Wei L; Chen Y; Ruan XZ. CD36 regulates diurnal glucose metabolism and hepatic clock to maintain glucose homeostasis in mice. iScience 2023, 26, 106524. 10.1016/j.isci.2023.106524. 37123238 PMC10139992

[87] Sinturel F; Gerber A; Mauvoisin D; Wang J; Gatfield D; Stubblefield JJ; Green CB; Gachon F; Schibler U. Diurnal Oscillations in Liver Mass and Cell Size Accompany Ribosome Assembly Cycles. Cell 2017, 169, 651–663.e614. 10.1016/j.cell.2017.04.015. 28475894 PMC5570523

[88] Wang J; Mauvoisin D; Martin E; Atger F; Galindo AN; Dayon L; Sizzano F; Palini A; Kussmann M; Waridel P; Nuclear Proteomics Uncovers Diurnal Regulatory Landscapes in Mouse Liver. Cell Metab. 2017, 25, 102–117. 10.1016/j.cmet.2016.10.003. 27818260 PMC5241201

[89] Mauvoisin D; Wang J; Jouffe C; Martin E; Atger F; Waridel P; Quadroni M; Gachon F; Naef F. Circadian clock-dependent and -independent rhythmic proteomes implement distinct diurnal functions in mouse liver. Proc. Natl. Acad. Sci. USA 2014, 111, 167–172, 10.1073/pnas.1314066111. 24344304 PMC3890886

[90] Kalhan SC; Ghosh A. Dietary Iron, Circadian Clock, and Hepatic Gluconeogenesis. Diabetes 2015, 64, 1091–1093. 10.2337/db14-1697. 25805759

[91] Hunter AL; Ray DW. Circadian Clock Regulation of Hepatic Energy Metabolism Regulatory Circuits. Biology 2019, 8, 79. 31635079 10.3390/biology8040079 PMC6956161

[92] Mukherji A; Bailey SM; Staels B; Baumert TF. The circadian clock and liver function in health and disease. J. Hepatol. 2019, 71, 200–211. 10.1016/j.jhep.2019.03.020. 30930223 PMC7613420

[93] Li H; Zhang S; Zhang W; Chen S; Rabearivony A; Shi Y; Liu J; Corton CJ; Liu C. Endogenous circadian time genes expressions in the liver of mice under constant darkness. BMC Genom. 2020, 21, 224. 10.1186/s12864-020-6639-4. PMC7066782 32160860

[94] Shulman GI. Ectopic fat in insulin resistance, dyslipidemia, and cardiometabolic disease. N. Engl. J. Med. 2014, 371, 1131–1141. 10.1056/NEJMra1011035. 25229917

[95] Carrasco-Benso MP; Rivero-Gutierrez B; Lopez-Minguez J; Anzola A; Diez-Noguera A; Madrid JA; Lujan JA; Martínez-Augustin O; Scheer FA; Garaulet M. Human adipose tissue expresses intrinsic circadian rhythm in insulin sensitivity. FASEB J. 2016, 30, 3117–3123. 10.1096/fj.201600269RR. 27256623 PMC5001513

[96] Hepler C; Bass J. Circadian mechanisms in adipose tissue bioenergetics and plasticity. Genes Dev. 2023, 37, 454–473. 10.1101/gad.350759.123. 37364987 PMC10393195

[97] Man AWC; Xia N; Li H. Circadian Rhythm in Adipose Tissue: Novel Antioxidant Target for Metabolic and Cardiovascular Diseases. Antioxidants 2020, 9, 968. 10.3390/antiox9100968. 33050331 PMC7601443

[98] Civelek E; Ozturk Civelek D; Akyel YK; Kaleli Durman D; Okyar A. Circadian Dysfunction in Adipose Tissue: Chronotherapy in Metabolic Diseases. Biology 2023, 12, 1077. 37626963 10.3390/biology12081077 PMC10452180

[99] Wang S; Lin Y; Gao L; Yang Z; Lin J; Ren S; Li F; Chen J; Wang Z; Dong Z; PPAR-γ integrates obesity and adipocyte clock through epigenetic regulation of Bmal1. Theranostics 2022, 12, 1589–1606. 10.7150/thno.69054. 35198059 PMC8825584

[100] Hasan N; Nagata N; Morishige J; Islam MT; Jing Z; Harada K.-i.; Mieda M; Ono M; Fujiwara H; Daikoku T; Brown adipocyte-specific knockout of Bmal1 causes mild but significant thermogenesis impairment in mice. Mol. Metab. 2021, 49, 101202. 10.1016/j.molmet.2021.101202. 33676029 PMC8042177

[101] Zvonic S; Ptitsyn AA; Conrad SA; Scott LK; Floyd ZE; Kilroy G; Wu X; Goh BC; Mynatt RL; Gimble JM. Characterization of peripheral circadian clocks in adipose tissues. Diabetes 2006, 55, 962–970. 10.2337/diabetes.55.04.06.db05-0873. 16567517

[102] Loboda A; Kraft WK; Fine B; Joseph J; Nebozhyn M; Zhang C; He Y; Yang X; Wright C; Morris M; Diurnal variation of the human adipose transcriptome and the link to metabolic disease. BMC Med. Genom. 2009, 2, 7. 10.1186/1755-8794-2-7. PMC2647943 19203388

[103] Shostak A; Meyer-Kovac J; Oster H. Circadian regulation of lipid mobilization in white adipose tissues. Diabetes 2013, 62, 2195–2203. 10.2337/db12-1449. 23434933 PMC3712056

[104] MacDougald OA; Burant CF. The rapidly expanding family of adipokines. Cell Metab. 2007, 6, 159–161. 10.1016/j.cmet.2007.08.010. 17767903

[105] Norton L; Shannon C; Gastaldelli A; DeFronzo RA. Insulin: The master regulator of glucose metabolism. Metabolism 2022, 129, 155142. 10.1016/j.metabol.2022.155142. 35066003

[106] James DE; Stöckli J; Birnbaum MJ. The aetiology and molecular landscape of insulin resistance. Nat. Rev. Mol. Cell Biol. 2021, 22, 751–771. 10.1038/s41580-021-00390-6. 34285405

[107] Muoio DM; Newgard CB. Mechanisms of disease:Molecular and metabolic mechanisms of insulin resistance and beta-cell failure in type 2 diabetes. Nat. Rev. Mol. Cell Biol. 2008, 9, 193–205. 10.1038/nrm2327. 18200017

[108] Leproult R; Holmbäck U; Van Cauter E. Circadian misalignment augments markers of insulin resistance and inflammation, independently of sleep loss. Diabetes 2014, 63, 1860–1869. 10.2337/db13-1546. 24458353 PMC4030107

[109] Morris CJ; Yang JN; Garcia JI; Myers S; Bozzi I; Wang W; Buxton OM; Shea SA; Scheer FA. Endogenous circadian system and circadian misalignment impact glucose tolerance via separate mechanisms in humans. Proc. Natl. Acad. Sci. USA 2015, 112, E2225–E2234. 10.1073/pnas.1418955112. 25870289 PMC4418873

[110] Windred DP; Burns AC; Rutter MK; Ching Yeung CH; Lane JM; Xiao Q; Saxena R; Cain SW; Phillips AJK. Personal light exposure patterns and incidence of type 2 diabetes: Analysis of 13 million hours of light sensor data and 670,000 person-years of prospective observation. Lancet Reg. Health Eur. 2024, 42, 100943. 10.1016/j.lanepe.2024.100943. 39070751 PMC11281921

[111] Lu H; Yang Q; Tian F; Lyu Y; He H; Xin X; Zheng X. A Meta-Analysis of a Cohort Study on the Association between Sleep Duration and Type 2 Diabetes Mellitus. J. Diabetes Res. 2021, 2021, 8861038. 10.1155/2021/8861038. 33834077 PMC8012145

[112] Her TK; Li J; Lin H; Liu D; Root KM; Regal JF; Alejandro EU; Cao R. Circadian Disruption across Lifespan Impairs Glucose Homeostasis and Insulin Sensitivity in Adult Mice. Metabolites 2024, 14, 126. 10.3390/metabo14020126. 38393018 PMC10892663

[113] Yamaguchi M; Uemura H; Arisawa K; Katsuura-Kamano S; Hamajima N; Hishida A; Suma S; Oze I; Nakamura K; Takashima N; Association between brain-muscle-ARNT-like protein-2 (BMAL2) gene polymorphism and type 2 diabetes mellitus in obese Japanese individuals: A cross-sectional analysis of the Japan Multi-institutional Collaborative Cohort Study. Diabetes Res. Clin. Pract. 2015, 110, 301–308. 10.1016/j.diabres.2015.10.009. 26497775

[114] Woon PY; Kaisaki PJ; Bragança J; Bihoreau MT; Levy JC; Farrall M; Gauguier D. Aryl hydrocarbon receptor nuclear translocator-like (BMAL1) is associated with susceptibility to hypertension and type 2 diabetes. Proc. Natl. Acad. Sci. USA 2007, 104, 14412–14417. 10.1073/pnas.0703247104. 17728404 PMC1958818

[115] Corella D; Asensio EM; Coltell O; Sorlí JV; Estruch R; Martínez-González M; Salas-Salvadó J; Castañer O; Arós F; Lapetra J; CLOCK gene variation is associated with incidence of type-2 diabetes and cardiovascular diseases in type-2 diabetic subjects: Dietary modulation in the PREDIMED randomized trial. Cardiovasc. Diabetol. 2016, 15, 4. 10.1186/s12933-015-0327-8. 26739996 PMC4704407

[116] Yoon M-S. The Role of Mammalian Target of Rapamycin (mTOR) in Insulin Signaling. Nutrients 2017, 9, 1176. 29077002 10.3390/nu9111176 PMC5707648

[117] Roux PP; Topisirovic I. Signaling Pathways Involved in the Regulation of mRNA Translation. Mol. Cell. Biol. 2018, 38, e00070–18. 10.1128/mcb.00070-18. 29610153 PMC5974435

[118] Lee H-S; Lee E; Moon J-H; Kim Y; Lee H-J. Circadian disruption and increase of oxidative stress in male and female volunteers after bright light exposure before bed time. Mol. Cell. Toxicol. 2019, 15, 221–229. 10.1007/s13273-019-0025-9.

[119] Boege HL; Bhatti MZ; St-Onge M-P. Circadian rhythms and meal timing: Impact on energy balance and body weight. Curr. Opin. Biotechnol. 2021, 70, 1–6. 10.1016/j.copbio.2020.08.009. 32998085 PMC7997809

[120] Solinas G; Becattini B. JNK at the crossroad of obesity, insulin resistance, and cell stress response. Mol. Metab. 2017, 6, 174–184. 10.1016/j.molmet.2016.12.001. 28180059 PMC5279903

[121] Catalano F; De Vito F; Cassano V; Fiorentino TV; Sciacqua A; Hribal ML. Circadian Clock Desynchronization and Insulin Resistance. Int. J. Environ. Res. Public Health 2022, 20, 29. 10.3390/ijerph20010029. 36612350 PMC9819930

[122] Karampelias C; Watt K; Mattsson CL; Ruiz ÁF; Rezanejad H; Mi J; Liu X; Chu L; Locasale JW; Korbutt GS; MNK2 deficiency potentiates β-cell regeneration via translational regulation. Nat. Chem. Biol. 2022, 18, 942–953. 10.1038/s41589-022-01047-x. 35697798 PMC7613404

[123] Lee J; Moulik M; Fang Z; Saha P; Zou F; Xu Y; Nelson DL; Ma K; Moore DD; Yechoor VK. Bmal1 and β-cell clock are required for adaptation to circadian disruption, and their loss of function leads to oxidative stress-induced β-cell failure in mice. Mol. Cell. Biol. 2013, 33, 2327–2338. 10.1128/mcb.01421-12. 23547261 PMC3648066

[124] Pekovic-Vaughan V; Gibbs J; Yoshitane H; Yang N; Pathiranage D; Guo B; Sagami A; Taguchi K; Bechtold D; Loudon A; The circadian clock regulates rhythmic activation of the NRF2/glutathione-mediated antioxidant defense pathway to modulate pulmonary fibrosis. Genes Dev. 2014, 28, 548–560. 10.1101/gad.237081.113. 24637114 PMC3967045

[125] Xu YQ; Zhang D; Jin T; Cai DJ; Wu Q; Lu Y; Liu J; Klaassen CD. Diurnal variation of hepatic antioxidant gene expression in mice. PLoS ONE 2012, 7, e44237. 10.1371/journal.pone.0044237. 22952936 PMC3430632

[126] Malhan D; Yalçin M; Schoenrock B; Blottner D; Relógio A. Skeletal muscle gene expression dysregulation in long-term spaceflights and aging is clock-dependent. npj Microgravity 2023, 9, 30. 10.1038/s41526-023-00273-4. 37012297 PMC10070655

[127] Lapehn S; Paquette AG. The Placental Epigenome as a Molecular Link Between Prenatal Exposures and Fetal Health Outcomes Through the DOHaD Hypothesis. Curr. Environ. Health Rep. 2022, 9, 490–501. 10.1007/s40572-022-00354-8. 35488174 PMC9363315

[128] Lecoutre S; Maqdasy S; Breton C. Maternal obesity as a risk factor for developing diabetes in offspring: An epigenetic point of view. World J. Diabetes 2021, 12, 366–382. 10.4239/wjd.v12.i4.366. 33889285 PMC8040079

[129] Yao N; Kinouchi K; Katoh M; Ashtiani KC; Abdelkarim S; Morimoto H; Torimitsu T; Kozuma T; Iwahara A; Kosugi S; Maternal circadian rhythms during pregnancy dictate metabolic plasticity in offspring. Cell Metab. 2025, 37, 395–412 e396. 10.1016/j.cmet.2024.12.002. 39814018 PMC11872692

[130] Bleker LS; de Rooij SR; Painter RC; Ravelli AC; Roseboom TJ. Cohort profile: The Dutch famine birth cohort (DFBC)- a prospective birth cohort study in the Netherlands. BMJ Open 2021, 11, e042078. 10.1136/bmjopen-2020-042078. PMC7934722 33664071

[131] Vetter C. Circadian disruption: What do we actually mean? Eur. J. Neurosci. 2020, 51, 531–550. 10.1111/ejn.14255. 30402904 PMC6504624

[132] Bates K; Herzog ED. Maternal-Fetal Circadian Communication During Pregnancy. Front. Endocrinol. 2020, 11, 198. 10.3389/fendo.2020.00198. PMC7174624 32351448

[133] McCarthy R; Jungheim ES; Fay JC; Bates K; Herzog ED; England SK. Riding the Rhythm of Melatonin Through Pregnancy to Deliver on Time. Front. Endocrinol. 2019, 10, 616. 10.3389/fendo.2019.00616. PMC6753220 31572299

[134] Reiter RJ; Tan DX; Korkmaz A; Rosales-Corral SA. Melatonin and stable circadian rhythms optimize maternal, placental and fetal physiology. Hum. Reprod. Update 2014, 20, 293–307. 10.1093/humupd/dmt054. 24132226

[135] Emet M; Ozcan H; Ozel L; Yayla M; Halici Z; Hacimuftuoglu A. A Review of Melatonin, Its Receptors and Drugs. Eurasian J. Med. 2016, 48, 135–141. 10.5152/eurasianjmed.2015.0267. 27551178 PMC4970552

[136] Sheynzon P; Karolczak M; Dehghani F; Korf HW. Diurnal variation in CREB phosphorylation and PER1 protein levels in lactotroph cells of melatonin-proficient C3H and melatonin-deficient C57BL mice: Similarities and differences. Cell Tissue Res. 2005, 321, 211–217. 10.1007/s00441-005-1150-4. 15947965

[137] Brenna A; Ripperger JA; Saro G; Glauser DA; Yang Z; Albrecht U. PER2 mediates CREB-dependent light induction of the clock gene Per1. Sci. Rep. 2021, 11, 21766. 10.1038/s41598-021-01178-6. 34741086 PMC8571357

[138] Rakshit K; Matveyenko AV. Induction of Core Circadian Clock Transcription Factor Bmal1 Enhances beta-Cell Function and Protects Against Obesity-Induced Glucose Intolerance. Diabetes 2021, 70, 143–154. 10.2337/db20-0192. 33087455 PMC7881843

[139] Okatani Y; Okamoto K; Hayashi K; Wakatsuki A; Tamura S; Sagara Y. Maternal-fetal transfer of melatonin in pregnant women near term. J. Pineal Res. 1998, 25, 129–134. 10.1111/j.1600-079x.1998.tb00550.x. 9745980

[140] Lanoix D; Beghdadi H; Lafond J; Vaillancourt C. Human placental trophoblasts synthesize melatonin and express its receptors. J. Pineal Res. 2008, 45, 50–60. 10.1111/j.1600-079X.2008.00555.x. 18312298

[141] Speirs HJ; Seckl JR; Brown RW. Ontogeny of glucocorticoid receptor and 11beta-hydroxysteroid dehydrogenase type-1 gene expression identifies potential critical periods of glucocorticoid susceptibility during development. J. Endocrinol. 2004, 181, 105–116. 10.1677/joe.0.1810105. 15072571

[142] Benediktsson R; Calder AA; Edwards CR; Seckl JR. Placental 11 beta-hydroxysteroid dehydrogenase: A key regulator of fetal glucocorticoid exposure. Clin. Endocrinol. 1997, 46, 161–166. 10.1046/j.1365-2265.1997.1230939.x. 9135697

[143] Mesiano S; Jaffe RB. Developmental and functional biology of the primate fetal adrenal cortex. Endocr. Rev. 1997, 18, 378–403. 10.1210/edrv.18.3.0304. 9183569

[144] Hsu CN; Tain YL. Light and Circadian Signaling Pathway in Pregnancy: Programming of Adult Health and Disease. Int. J. Mol. Sci. 2020, 21, 2232. 10.3390/ijms21062232. 32210175 PMC7139376

[145] Zhao B; Yu Z; Sun J; Cheng W; Yu T; Yang Y; Wei Z; Yin Z. Light pollution during pregnancy influences the growth of offspring in rats. Ecotoxicol. Environ. Saf. 2024, 279, 116485. 10.1016/j.ecoenv.2024.116485. 38788564

[146] Benediktsson R; Lindsay RS; Noble J; Seckl JR; Edwards CR. Glucocorticoid exposure in utero: New model for adult hypertension. Lancet 1993, 341, 339–341. 10.1016/0140-6736(93)90138-7. 8094115

[147] Seckl JR. 11beta-hydroxysteroid dehydrogenases: Changing glucocorticoid action. Curr. Opin. Pharmacol. 2004, 4, 597–602. 10.1016/j.coph.2004.09.001. 15525550

[148] Nyirenda MJ; Lindsay RS; Kenyon CJ; Burchell A; Seckl JR. Glucocorticoid exposure in late gestation permanently programs rat hepatic phosphoenolpyruvate carboxykinase and glucocorticoid receptor expression and causes glucose intolerance in adult offspring. J. Clin. Investig. 1998, 101, 2174–2181. 10.1172/JCI1567. 9593773 PMC508805

[149] Yu CY; Mayba O; Lee JV; Tran J; Harris C; Speed TP; Wang JC. Genome-wide analysis of glucocorticoid receptor binding regions in adipocytes reveal gene network involved in triglyceride homeostasis. PLoS ONE 2010, 5, e15188. 10.1371/journal.pone.0015188. 21187916 PMC3004788

[150] Phan J; Reue K. Lipin, a lipodystrophy and obesity gene. Cell Metab. 2005, 1, 73–83. 10.1016/j.cmet.2004.12.002. 16054046

[151] Ding L; Weger BD; Liu J; Zhou L; Lim Y; Wang D; Xie Z; Liu J; Ren J; Zheng J; Maternal high fat diet induces circadian clock-independent endocrine alterations impacting the metabolism of the offspring. iScience 2024, 27, 110343. 10.1016/j.isci.2024.110343. 39045103 PMC11263959

[152] Kawai M; Green CB; Lecka-Czernik B; Douris N; Gilbert MR; Kojima S; Ackert-Bicknell C; Garg N; Horowitz MC; Adamo ML; A circadian-regulated gene, Nocturnin, promotes adipogenesis by stimulating PPAR-gamma nuclear translocation. Proc. Natl. Acad. Sci. USA 2010, 107, 10508–10513. 10.1073/pnas.1000788107. 20498072 PMC2890788

[153] Mendez N; Halabi D; Salazar-Petres ER; Vergara K; Corvalan F; Richter HG; Bastidas C; Bascur P; Ehrenfeld P; Seron-Ferre M; Maternal melatonin treatment rescues endocrine, inflammatory, and transcriptional deregulation in the adult rat female offspring from gestational chronodisruption. Front. Neurosci. 2022, 16, 1039977. 10.3389/fnins.2022.1039977. 36507347 PMC9727156

[154] Lee J; Kim MS; Li R; Liu VY; Fu L; Moore DD; Ma K; Yechoor VK. Loss of Bmal1 leads to uncoupling and impaired glucose-stimulated insulin secretion in beta-cells. Islets 2011, 3, 381–388. 10.4161/isl.3.6.18157. 22045262 PMC3329519

[155] Mendez N; Halabi D; Spichiger C; Salazar ER; Vergara K; Alonso-Vasquez P; Carmona P; Sarmiento JM; Richter HG; Seron-Ferre M; Gestational Chronodisruption Impairs Circadian Physiology in Rat Male Offspring, Increasing the Risk of Chronic Disease. Endocrinology 2016, 157, 4654–4668. 10.1210/en.2016-1282. 27802074

[156] Desai M; Ferrini MG; Han G; Narwani K; Ross MG. Maternal High Fat Diet Programs Male Mice Offspring Hyperphagia and Obesity: Mechanism of Increased Appetite Neurons via Altered Neurogenic Factors and Nutrient Sensor AMPK. Nutrients 2020, 12, 3326. 10.3390/nu12113326. 33138074 PMC7693487

[157] Maske CB; Jackson CM; Terrill SJ; Eckel LA; Williams DL. Estradiol modulates the anorexic response to central glucagon-like peptide 1. Horm. Behav. 2017, 93, 109–117. 10.1016/j.yhbeh.2017.05.012. 28558993 PMC5555302

[158] Eckel LA; Geary N. Estradiol treatment increases feeding-induced c-Fos expression in the brains of ovariectomized rats. Am. J. Physiol. Regul. Integr. Comp. Physiol. 2001, 281, R738–R746. 10.1152/ajpregu.2001.281.3.R738. 11506987

[159] Nugent BM; O’Donnell CM; Epperson CN; Bale TL. Placental H3K27me3 establishes female resilience to prenatal insults. Nat. Commun. 2018, 9, 2555. 10.1038/s41467-018-04992-1. 29967448 PMC6028627

[160] Olivier-Van Stichelen S; Abramowitz LK; Hanover JA. X marks the spot: Does it matter that O-GlcNAc transferase is an X-linked gene? Biochem. Biophys. Res. Commun. 2014, 453, 201–207. 10.1016/j.bbrc.2014.06.068. 24960196 PMC4253714

[161] Kuriyama K; Sasahara K; Kudo T; Shibata S. Daily injection of insulin attenuated impairment of liver circadian clock oscillation in the streptozotocin-treated diabetic mouse. FEBS Lett. 2004, 572, 206–210. 10.1016/j.febslet.2004.07.036. 15304349

[162] Türk D; Scherer N; Selzer D; Dings C; Hanke N; Dallmann R; Schwab M; Timmins P; Nock V; Lehr T. Significant impact of time-of-day variation on metformin pharmacokinetics. Diabetologia 2023, 66, 1024–1034. 10.1007/s00125-023-05898-4. 36930251 PMC10163090

[163] Thomas AP; Hoang J; Vongbunyong K; Nguyen A; Rakshit K; Matveyenko AV. Administration of Melatonin and Metformin Prevents Deleterious Effects of Circadian Disruption and Obesity in Male Rats. Endocrinology 2016, 157, 4720–4731. 10.1210/en.2016-1309. 27653034 PMC5133345

[164] Hirota T; Lee JW; St John PC; Sawa M; Iwaisako K; Noguchi T; Pongsawakul PY; Sonntag T; Welsh DK; Brenner DA; Identification of small molecule activators of cryptochrome. Science 2012, 337, 1094–1097. 10.1126/science.1223710. 22798407 PMC3589997

[165] Kumar N; Solt LA; Wang Y; Rogers PM; Bhattacharyya G; Kamenecka TM; Stayrook KR; Crumbley C; Floyd ZE; Gimble JM; Regulation of adipogenesis by natural and synthetic REV-ERB ligands. Endocrinology 2010, 151, 3015–3025. 10.1210/en.2009-0800. 20427485 PMC2903944

[166] Marciano DP; Chang MR; Corzo CA; Goswami D; Lam VQ; Pascal BD; Griffin PR. The Therapeutic Potential of Nuclear Receptor Modulators for Treatment of Metabolic Disorders: PPARγ, RORs, and Rev-erbs. Cell Metab. 2014, 19, 193–208. 10.1016/j.cmet.2013.12.009. 24440037

[167] He B; Nohara K; Park N; Park YS; Guillory B; Zhao Z; Garcia JM; Koike N; Lee CC; Takahashi JS; The Small Molecule Nobiletin Targets the Molecular Oscillator to Enhance Circadian Rhythms and Protect against Metabolic Syndrome. Cell Metab. 2016, 23, 610–621. 10.1016/j.cmet.2016.03.007. 27076076 PMC4832569

[168] Li X; Zhuang R; Lu Z; Wu F; Wu X; Zhang K; Wang M; Li W; Zhang H; Zhu W; Nobiletin promotes lipolysis of white adipose tissue in a circadian clock-dependent manner. J. Nutr. Biochem. 2024, 132, 109696. 10.1016/j.jnutbio.2024.109696. 39094217

[169] Surme S; Ergun C; Gul S; Akyel YK; Gul ZM; Ozcan O; Ipek OS; Akarlar BA; Ozlu N; Taskin AC; TW68, cryptochromes stabilizer, regulates fasting blood glucose levels in diabetic ob/ob and high fat-diet-induced obese mice. Biochem. Pharmacol. 2023, 218, 115896. 10.1016/j.bcp.2023.115896. 37898388

[170] Qi G; Guo R; Tian H; Li L; Liu H; Mi Y; Liu X. Nobiletin protects against insulin resistance and disorders of lipid metabolism by reprogramming of circadian clock in hepatocytes. Biochim. Biophys. Acta (BBA)—Mol. Cell Biol. Lipids 2018, 1863, 549–562. 10.1016/j.bbalip.2018.02.009. 29501626

[171] Mi Y; Qi G; Gao Y; Li R; Wang Y; Li X; Huang S; Liu X. (−)-Epigallocatechin-3-gallate Ameliorates Insulin Resistance and Mitochondrial Dysfunction in HepG2 Cells: Involvement of Bmal1. Mol. Nutr. Food Res. 2017, 61, 1700440. 10.1002/mnfr.201700440. 28869341

[172] Manjula A; Subashini R; Punitha R; Subramanian P. Modulating effects of hesperidin on circadian pattern indices of rotenone induced redox homeostasis in clock mutant (cryb) of Drosophila melanogaster. Biol. Rhythm. Res. 2017, 48, 897–906. 10.1080/09291016.2017.1319641.

[173] Zhou L; Xiao X; Zhang Q; Zheng J; Li M; Yu M; Wang X; Deng M; Zhai X; Li R; Dietary Genistein Could Modulate Hypothalamic Circadian Entrainment, Reduce Body Weight, and Improve Glucose and Lipid Metabolism in Female Mice. Int. J. Endocrinol. 2019, 2019, 2163838. 10.1155/2019/2163838. 31139215 PMC6500629

[174] Jiang J; Gu Y; Ding S; Zhang G; Ding J. Resveratrol reversed ambient particulate matter exposure-perturbed oscillations of hepatic glucose metabolism by regulating SIRT1 in mice. Environ. Sci. Pollut. Res. 2023, 30, 31821–31834. 10.1007/s11356-022-24434-2. 36459324

